# Impact of asthma on the brain: evidence from diffusion MRI, CSF biomarkers and cognitive decline

**DOI:** 10.1093/braincomms/fcad180

**Published:** 2023-06-13

**Authors:** Ajay Kumar Nair, Carol A Van Hulle, Barbara B Bendlin, Henrik Zetterberg, Kaj Blennow, Norbert Wild, Gwendlyn Kollmorgen, Ivonne Suridjan, William W Busse, Douglas C Dean, Melissa A Rosenkranz

**Affiliations:** Center for Healthy Minds, University of Wisconsin-Madison, Madison, WI 53703, USA; Wisconsin Alzheimer’s Disease Research Center, School of Medicine and Public Health, University of Wisconsin-Madison, Madison, WI 53792, USA; Department of Medicine, School of Medicine and Public Health, University of Wisconsin-Madison, Madison, WI 53792, USA; Wisconsin Alzheimer’s Disease Research Center, School of Medicine and Public Health, University of Wisconsin-Madison, Madison, WI 53792, USA; Department of Medicine, School of Medicine and Public Health, University of Wisconsin-Madison, Madison, WI 53792, USA; Wisconsin Alzheimer’s Institute, School of Medicine and Public Health, University of Wisconsin-Madison, Madison, WI 53726, USA; Department of Psychiatry and Neurochemistry, Institute of Neuroscience and Physiology, the Sahlgrenska Academy at the University of Gothenburg, S-431 30 Mölndal, Sweden; Clinical Neurochemistry Laboratory, Sahlgrenska University Hospital, S-431 30 Mölndal, Sweden; Department of Neurodegenerative Disease, UCL Institute of Neurology, London, WC1N 3BG, UK; UK Dementia Research Institute at UCL, London, WCIE 6BT, UK; Hong Kong Center for Neurodegenerative Diseases, Hong Kong, Clear Water Bay, Hong Kong SAR, China; Department of Psychiatry and Neurochemistry, Institute of Neuroscience and Physiology, the Sahlgrenska Academy at the University of Gothenburg, S-431 30 Mölndal, Sweden; Clinical Neurochemistry Laboratory, Sahlgrenska University Hospital, S-431 30 Mölndal, Sweden; Roche Diagnostics GmbH, Core Lab RED, 82377 Penzberg, Germany; Roche Diagnostics GmbH, Clinical Operations, 82377 Penzberg, Germany; CDMA Clinical Development, Roche Diagnostics International Ltd, CH-6346, Rotkreuz, Switzerland; Department of Medicine, School of Medicine and Public Health, University of Wisconsin-Madison, Madison, WI 53792, USA; Department of Pediatrics, University of Wisconsin School of Medicine and Public Health, Madison, WI 53792, USA; Department of Medical Physics, University of Wisconsin School of Medicine and Public Health, Madison, WI 53705, USA; Waisman Center, University of Wisconsin-Madison, Madison, WI 53705, USA; Center for Healthy Minds, University of Wisconsin-Madison, Madison, WI 53703, USA; Department of Psychiatry, University of Wisconsin-Madison, Madison, WI 53719, USA

**Keywords:** asthma, dementia, cognition, CSF biomarkers, diffusion MRI

## Abstract

Chronic systemic inflammation increases the risk of neurodegeneration, but the mechanisms remain unclear. Part of the challenge in reaching a nuanced understanding is the presence of multiple risk factors that interact to potentiate adverse consequences. To address modifiable risk factors and mitigate downstream effects, it is necessary, although difficult, to tease apart the contribution of an individual risk factor by accounting for concurrent factors such as advanced age, cardiovascular risk, and genetic predisposition.

Using a case-control design, we investigated the influence of asthma, a highly prevalent chronic inflammatory disease of the airways, on brain health in participants recruited to the Wisconsin Alzheimer’s Disease Research Center (31 asthma patients, 186 non-asthma controls, aged 45–90 years, 62.2% female, 92.2% cognitively unimpaired), a sample enriched for parental history of Alzheimer’s disease. Asthma status was determined using detailed prescription information. We employed multi-shell diffusion weighted imaging scans and the three-compartment neurite orientation dispersion and density imaging model to assess white and gray matter microstructure. We used cerebrospinal fluid biomarkers to examine evidence of Alzheimer’s disease pathology, glial activation, neuroinflammation and neurodegeneration. We evaluated cognitive changes over time using a preclinical Alzheimer cognitive composite. Using permutation analysis of linear models, we examined the moderating influence of asthma on relationships between diffusion imaging metrics, CSF biomarkers, and cognitive decline, controlling for age, sex, and cognitive status. We ran additional models controlling for cardiovascular risk and genetic risk of Alzheimer’s disease, defined as a carrier of at least one apolipoprotein E (*APOE*) *ε*4 allele.

Relative to controls, greater Alzheimer’s disease pathology (lower amyloid-β_42_/amyloid-β_40_, higher phosphorylated-tau-181) and synaptic degeneration (neurogranin) biomarker concentrations were associated with more adverse white matter metrics (e.g. lower neurite density, higher mean diffusivity) in patients with asthma. Higher concentrations of the pleiotropic cytokine IL-6 and the glial marker S100B were associated with more salubrious white matter metrics in asthma, but not in controls. The adverse effects of age on white matter integrity were accelerated in asthma. Finally, we found evidence that in asthma, relative to controls, deterioration in white and gray matter microstructure was associated with accelerated cognitive decline.

Taken together, our findings suggest that asthma accelerates white and gray matter microstructural changes associated with aging and increasing neuropathology, that in turn, are associated with more rapid cognitive decline. Effective asthma control, on the other hand, may be protective and slow progression of cognitive symptoms.

## Teaser

Nair *et al.* report that changes in white matter microstructure associated with aging and increasing concentrations of CSF biomarkers of synaptic degeneration and Alzheimer’s disease pathology are accelerated in patients with asthma, relative to controls. Further, these deteriorations in brain health were associated with steeper cognitive decline in asthma.

## Graphical Abstract

**Graphical abstract:**
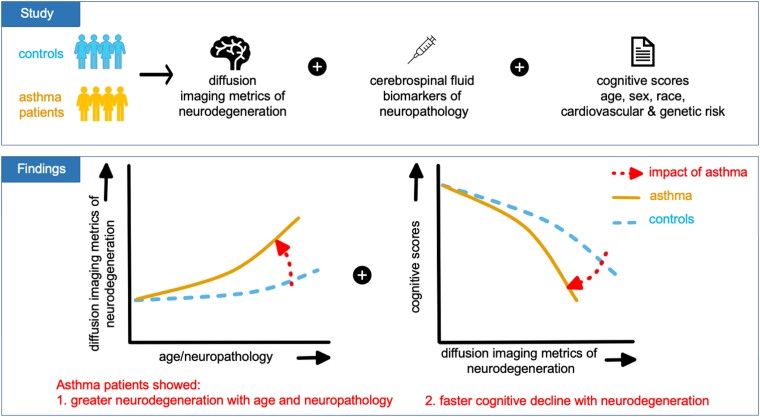

## Introduction

Systemic inflammation contributes to neurodegeneration via changes in vasculature and the development of a neuroinflammatory profile.^1,2^ These deleterious effects are associated with accelerated cognitive decline and heightened risk of progression to Alzheimer’s disease.^1^ Among those with Alzheimer’s disease, acute episodes of systemic inflammation are associated with increased neuroinflammation and accelerated cognitive decline, and the impact is higher in those with chronic inflammation.^3,4^ At the same time, it is well-recognized that neuropathological changes appear decades before clinical manifestations of dementia, with multiple pathways determining dementia prognosis.^5,6^ Thus, among those at risk for dementia, chronic inflammatory conditions magnify the risk and present an opportunity for effective intervention strategies to delay, if not prevent, the onset of dementia for these individuals.^7^

Increasing evidence points to the presence of a lung-brain axis, wherein airway physiology impacts brain structure and function.^8–10^ In asthma, a highly prevalent chronic inflammatory disease characterized by reversible airway hyper-responsiveness,^11^ a growing body of evidence indicates bidirectional lung-brain communication.^12–17^ Several epidemiological studies have found that asthma increases the risk of cognitive impairment and dementia^18–20^ suggesting that the lung-brain axis might be involved. There are, however, some studies that have found mixed or no evidence of the deleterious impact of asthma on brain health,^21–23^ which highlights multiple challenges in determining the contribution of a particular inflammatory condition to dementia risk, such as the presence of other co-morbid conditions that might accentuate risk^7^ or the potential protective effect of anti-inflammatory medications.^2,9^ Although these challenges are difficult to fully address in an ecologically valid way, convergent evidence from multiple indicators of neuropathology can provide more definitive evidence of the effects of asthma and delineate the scope of its adverse impact on the brain.

We recently reported evidence of widespread deleterious white matter changes, coupled with plasma biomarker evidence of neuroinflammation and neurodegeneration in individuals with asthma.^24^ In a separate study, we examined a panel of dementia-relevant CSF biomarkers and found that severe asthma is associated with synaptic degeneration and accelerated cognitive decline.^25^ The dominant model for disease progression in Alzheimer’s disease proposes that amyloid-β oligomerization in limbic and association cortices triggers glial activity and is followed by oxidative stress, alterations in kinase and phosphatase activity, formation of neurofibrillary tangles, synaptic dysfunction, and neuronal loss leading to dementia.^26^ In the presence of co-morbid pathologies, the order of disease progression could change (for example tauopathy may precede amyloid deposition) but most models suggest that neuropathological changes typically precede neurodegenerative changes that can be detected by MRI, which in turn precedes cognitive impairment;^5,27^ we hypothesized that asthma would amplify this progression. A direct examination of the influence of asthma on white and gray matter microstructure and CSF biomarkers associated with dementia, in the same patients, has not yet been performed. Such an investigation would make it possible to determine if, and to what extent, asthma accelerates the impact of neuropathology (as suggested by specific CSF biomarkers) on neurodegenerative processes (assessed via diffusion imaging) and promotes cognitive decline. It would also enable determination of the spatial specificity versus global nature of these interactions. If asthma does accelerate cognitive decline, effective asthma management may present an opportunity to reduce the associated personal, societal, and healthcare burden.

Recent advances in diffusion neuroimaging (dMRI) allow detailed characterization of both white matter and gray matter microstructure *in vivo*.^28,29^ Traditional dMRI methods such as diffusion tensor imaging (DTI), provide a range of metrics that offer valuable information (fractional anisotropy, mean, radial, and axial diffusivity), but have limitations in their biological inference.^30^ Using multi-shell diffusion imaging, the three-compartment neurite orientation dispersion and density imaging (NODDI) model can be fit at each voxel to derive tissue microstructural information based on restricted diffusion in intracellular space (within neurites, i.e. axons and dendrites), hindered extracellular diffusion (near neurites, such as within neuronal soma and within glia), and free diffusion (such as in CSF).^29^

Developments in CSF biomarkers have also yielded a range of approaches that can be used to assess deleterious brain changes that emerge before clinical symptoms are detected.^5,31^ These CSF biomarkers index glial activation, neuroinflammation, synaptic and axonal degeneration, as well as Alzheimer’s disease pathology, such as reduced CSF concentration of the 42 amino acid-long amyloid-β protein (Aβ42) and elevated CSF phosphorylated tau concentration.^32^ Given evidence from animal models of asthma, demonstrating that allergen provocation in sensitized mice leads to microglial activation, dendritic atrophy and neuronal loss,^9,33^ examination of these biomarkers in asthma patients is essential.

Based on the literature showing exacerbating effects of systemic inflammation on cognitive decline,^2–4^ in the present study, we tested the hypothesis that chronic airway inflammation due to asthma is associated with neuroinflammation and neurodegeneration and accelerates progression on the Alzheimer’s continuum. To this end, we examined dMRI metrics, a panel of biomarkers measured in CSF and a longitudinal cognitive composite measure in an aging, mostly cognitively unimpaired (CU), sample enriched for parental history of Alzheimer’s disease.

## Materials and methods

### Selection of participants

Participants aged 45–90 years were recruited as part of an ongoing series of studies by the Wisconsin Alzheimer’s Disease Research Center (ADRC) and underwent multi-shell diffusion weighted (DWI) MRI. The sample was enriched for Alzheimer’s disease risk based on parental history. Participants with asthma were determined based on the National Institutes of Health (NIH) Guidelines for Asthma Diagnosis and Management,^34^ using detailed information regarding physician-prescribed asthma medication. The present study included only those participants with DWI and cognitive composite data, regardless of cognitive status, who underwent lumbar puncture for collection of CSF samples (total *n* = 217; *n* = 31 with asthma and *n* = 186 controls without asthma or other chronic respiratory diseases). Exclusion criteria included major medical, neurological, or psychiatric conditions. Participants with any history of major CNS comorbidities or other systemic inflammatory conditions (see Supplementary material for details) were excluded from analyses. All participants provided written informed consent in accordance with the declaration of Helsinki, and as approved (# 2013-0178) by the University of Wisconsin institutional review board.

Prescription medications for asthma included short- and long-acting bronchodilators, inhaled corticosteroids, systemic corticosteroids in conjunction with other asthma medication, and/or biologics specific for asthma. A complete list of asthma medications is available in the Supplementary material. Participant-reported diagnosis was used to confirm asthma status when possible.

### Neuroimaging

#### MRI acquisition

MRI data were acquired using a General Electric 3 Tesla MR750 scanner (Waukesha, WI) with a 32-channel head coil. DWIs were acquired using a multi-shell spin-echo echo-planar imaging pulse sequence with three diffusion encoding strengths (*b*-values) comprising 9 directions with *b* = 500 s/mm^2^, 18 directions with *b* = 800 s/mm^2^, and 36 directions with *b* = 2000 s/mm^2^, in addition to six images acquired without any diffusion encoding (*b* = 0 s/mm^2^). Other acquisition parameters included repetition time (TR) = 8575 ms, echo time (TE) = 76.8 ms; flip angle = 90°; and voxel resolution of 2 × 2 × 2 mm^3^ using a 128 × 128 acquisition matrix and 74 slices. The total acquisition time was approximately 10 minutes. Structural (T1-weighted) scans were acquired using a 3D inversion recovery prepared fast spoiled gradient-echo BRAVO sequence with inversion time = 450 ms; TR = 6.68 ms; TE = 2.94 ms; flip angle = 12°; 1 × 1 × 1 mm^3^ voxel resolution; and 256 × 256 acquisition matrix. The T1-weighted and diffusion-weighted MRI scans were acquired during the same imaging session in all participants as part of a larger standardized data collection protocol.

DWIs were processed using in-house pipelines. Briefly, images were denoised^35^ and corrected for Gibb’s ringing^36^ using MRtrix3 software^37^ and then corrected for movement and eddy current distortion using the ‘eddy’ tool^38,39^ in FSL (v6.0.4).^40^ Participant head motion during the DWI acquisition was quantitatively evaluated using the root mean square movement summary from eddy and outlier images were removed using ‘eddy-quad’.^41^ No participants were excluded from analyses based on visual inspection or excessive motion. Diffusion data were co-registered to an anatomical T1-weighted image for correcting distortions due to echo planar imaging^42^ and a brain mask was constructed using FSL’s Brain Extraction Tool.^43^ Diffusion tensors were fit at each voxel within the brain mask using the Diffusion Imaging in Python (DIPY) software package^44^ using weighted least-squares optimization. Voxelwise estimates of fractional anisotropy (FA) and axial (AD), radial (RD), and mean (MD) diffusivity were subsequently generated.^45,46^ Additionally, the three-compartment NODDI model^29^ was fit using the Diffusion Microstructure Imaging in Python (DMIPY) software^47^ to provide voxelwise estimates of the neurite density index (NDI), corresponding to the intra-cellular volume fraction, orientation dispersion index (ODI), and the isotropic volume fraction (FISO). The NODDI metrics offer more biologically interpretable information. For example, lower NDI is suggestive of degenerative changes in axons^48^ whereas higher ODI is suggestive of axonal disorganization in white matter.^49^ All images were visually inspected for quality and FA images from 100 participants were chosen to create a population-specific template using the *antsMultivariateTemplateConstruction2.sh* script from the Advanced Normalization Tools software package (ANTs v2.3.3).^50^ The population-specific template was registered to a 2 × 2 × 2 mm FA map provided by FSL to generate a final template. Individual parameter maps were registered and transformed to the final template space. Each set of parameter maps were then merged, and the corresponding FA skeleton map from FSL was used as a mask for voxelwise Tract-based Spatial Statistics (TBSS).^51^

**Figure 1:**
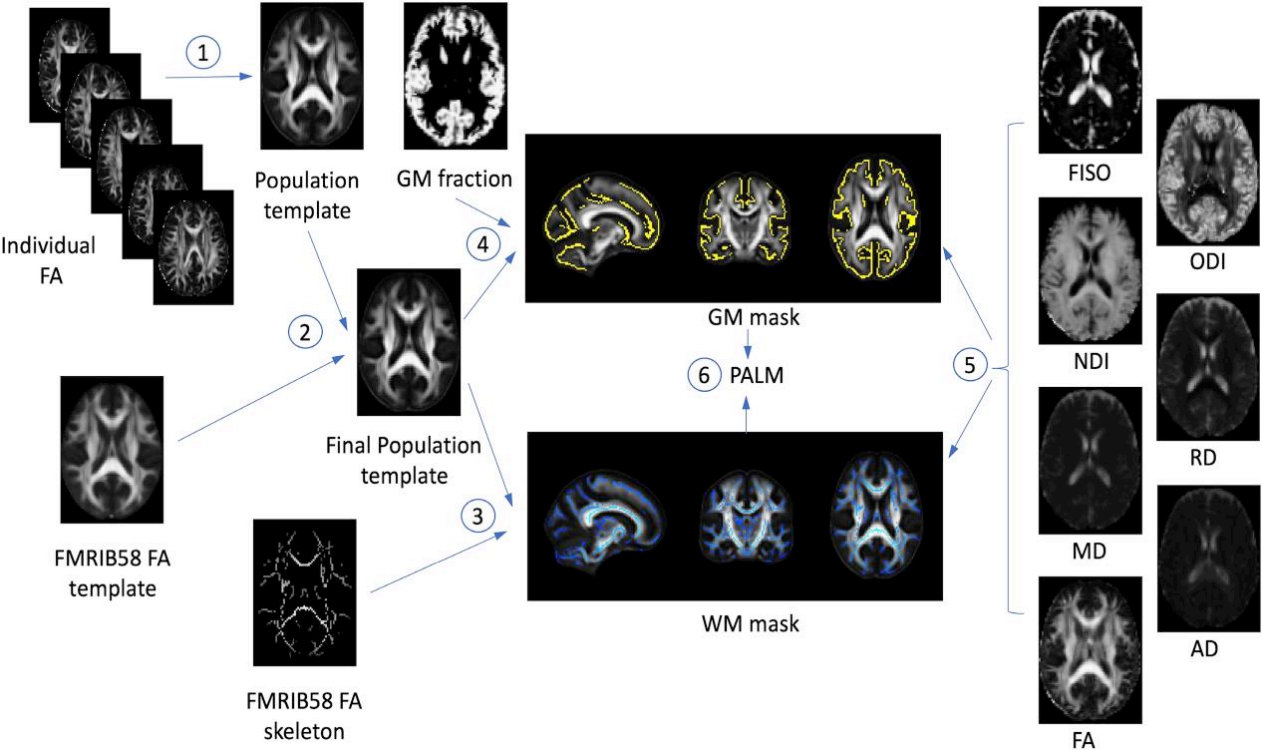
**Overview of diffusion image processing pipeline.** Initially, diffusion tensors were fit at every voxel to generate FA, AD, MD, RD maps for each participant. The three-compartment NODDI model was also fit at each voxel to generate NDI, ODI, and FISO maps. 1. Individual FA maps from 100 participants were used to create a population template using *antsMultivariateTemplateConstruction2.sh* script from ANTs. 2. This template was coregistered to the FMRIB58_FA template provided by FSL and transformed using the generated warp fields to arrive at the final population template. For each individual, all seven diffusion MRI parameter maps as well as the GM fraction maps were transformed to the final template space. 3. The FMRIB58_FA skeleton mask was used as the WM mask with the final template. 4. The GM fraction maps were used to create a mean skeletonized image using *tbss_skeleton* tool from FSL and a threshold set to only include voxels with GM fraction >0.65 in at least 70% of the participants. This GM skeletonized image was used as the GM mask in statistical analyses. 5. Individual maps in population space were merged for analyses restricted to the WM and GM masks. 6. Voxelwise analyses were carried out using PALM. AD, axial diffusivity; ANTs, advanced normalization tools; FA, fractional anisotropy; FISO, isotropic volume fraction; FSL, FMRIB software library; GM, gray matter; MD, mean diffusivity; NDI, neurite density index; NODDI, neurite orientation dispersion and density imaging; ODI, orientation dispersion index; PALM, permutation analysis of linear models; RD, radial diffusivity; WM, white matter.

To carry out voxelwise Gray-matter Based Spatial Statistics (GBSS),^52,53^ white matter fraction maps were first estimated from FA maps using the *Atropos* segmentation tool.^54^ Following procedures in Nazeri *et al.* 2015, gray matter fraction maps were then generated by subtracting the white matter and CSF fraction maps from a binarized brain mask.^52^ The gray matter fraction maps were also nonlinearly warped to the final template and averaged to create a mean gray matter image, which was then skeletonized using FSL’s *tbss_skeleton* tool and thresholded to only include voxels with gray matter fraction >0.65 in >70% of participants.^53^ Finally, DTI and NODDI parameter maps were projected onto the gray matter skeleton for statistical analysis. Figure 1 provides an overview of the processing steps after fitting the diffusion tensors and NODDI model.

#### Cerebrospinal fluid assessments

CSF samples (22 mL) were collected, centrifuged, and 0.5 mL CSF was aliquoted into 1.5 mL polypropylene tubes for storage at -80°C within 30 minutes of collection. The prototype NeuroToolKit (NTK; Roche Diagnostics International Ltd, Rotkreuz, Switzerland) assay panel of CSF biomarkers was used. The panel includes biomarkers for glial activation and inflammation: chitinase-3-like protein 1 (YKL-40), glial fibrillary acidic protein (GFAP), S100 calcium-binding protein B (S100B), soluble triggering receptor expressed on myeloid cells 2 (sTREM2), and interleukin-6 (IL-6); biomarkers of synaptic damage: α-synuclein and neurogranin; a biomarker of axonal degeneration: neurofilament light (NfL) protein; and biomarkers of Alzheimer’s disease pathology: Aβ40, Aβ42, and phosphorylated-tau-181 (Phospho-tau(181P)). The ratio Aβ42/Aβ40 was used in analyses.^31^ Concentrations of the Alzheimer’s disease pathology markers are well-studied and interpretable, whereas the roles of some of the other markers are less straightforward. For example, the astrocytic marker S100B, and the inflammatory cytokine IL-6 are known to be pleiotropic, changing their roles from physiological to neurotoxic based on concentrations.^55,56^ The cobas e 601 and cobas e 411 analyzers (Roche Diagnostics International Ltd, Rotkreuz, Switzerland) were used to perform immunoassays at the clinical neurochemistry laboratory, University of Gothenburg, following strict quality control procedures. The coefficient of variation for the assays were in the range of 1–3%, except for S100B (9%).

#### Cognitive assessments

A challenge with pre-clinical cognitive assessments is that individual scores fluctuate across repeated measurements and the separate use of several measures increases the risk of spuriously inflating the detection of cognitive impairment.^57^ As a result, use of comprehensive neuropsychological assessments, combined into a single composite measure is recommended to improve reliability and has been shown to track biomarker data.^57,58^ Accordingly, preclinical Alzheimer cognitive composite (PACC)^57,59^ scores were computed with equal weights given to the Rey Auditory Verbal Learning Task (total over five trials),^60^ Logical Memory IIA (Story Recall Delayed or cross-walked Craft Story),^61,62^ Trail-Making Test Part B,^63^ and the Mini-Mental State Examination or cross-walked Montreal Cognitive Assessment.^62,64^ Cognitive status was determined at a consensus review. Diagnoses of mild cognitive impairment or dementia were assigned based on National Institute on Aging-Alzheimer's Association (NIA-AA) criteria,^65,66^ without reference to biomarkers. Cognitive testing was performed annually for those with cognitive impairments and for CU participants aged 65 years or older, and biennially for CU participants aged under 65 years. Participants completed up to nine visits for cognitive assessments (*M* = 4.72, SD = 1.73). The computed PACC scores were z-transformed based on the distribution of data from a larger pool of all ADRC participants (*n* = 922). To evaluate changes in cognition over time, PACC slopes were derived from a linear mixed effects model.^32^

#### Pre-existing risk factors

To better isolate the influence of asthma on the brain, pre-existing risk factors for neurodegeneration were considered. *APOE* genotype was known for most participants (total *n* = 192, asthma *n* = 30). Those who carry at least one *APOE* ε4 allele (*APOE*4+) are at higher risk for Alzheimer’s disease.^67^ The 10-year sex- and race-specific risk for atherosclerotic cardiovascular disease (ASCVD) was calculated for these participants at study entry as described previously.^32,68^

### Statistical analyses

Permutation analysis of linear models (PALM)^69,70^ was used to examine the overall influence of asthma on dMRI metrics on a voxelwise basis, as well as the moderating influence of asthma on the relationship between dMRI metrics and CSF biomarkers, age, and cognitive decline. These whole-brain analyses were restricted to white matter and gray matter skeletons as described above, and were run using tail acceleration and 500 permutations.^69^ Joint inference over all seven dMRI metrics (FA, MD, RD, AD, NDI, ODI, and FISO), which we refer to as an ‘omnibus test’, was carried out using non-parametric combination (NPC) and Fisher’s combining function, and the inference about the contribution of each metric was obtained simultaneously. Evaluation of the relationship of each dMRI metric with age included sex and cognitive status as covariates. All other analyses included age, sex, and cognitive status as covariates. Analyses of dMRI data in relation to PACC slopes additionally controlled for baseline PACC scores. To parse the influence of existing cardiovascular risk or genetic risk for Alzheimer’s disease from the influence of asthma on the relationship between brain microstructure and CSF biomarkers, analyses were carried out including ASCVD and *APOE*4 genotype separately and jointly as additional covariates. Participants with missing ASCVD and *APOE*4 data (asthma 1, control 24) were dropped from analyses with these covariates. Threshold-free cluster enhancement and family-wise error (FWE) correction were used to control inflation of type I error.^71^ Voxels showing significant associations with regressors of interest were defined as those with *P<* 0.05, corrected for multiple comparisons and were visualized by thresholding statistical maps. All reported effects, unless otherwise mentioned, were significant in the omnibus test across all seven dMRI metrics. Data from voxels with median values of the t-statistic were averaged for each participant to derive summary estimates of the interaction coefficients. To visualize significant associations, mean values across significant voxels were extracted for each participant and the relationships of each dMRI metric with the variable of interest were plotted after removing influential model outliers that exceeded a threshold of 10% of the F-distribution of Cook’s distance. Visualizations were carried out in R statistical software (v3.6.1).^72^

## Results

### Study participants

Participant characteristics are presented in Table 1. Participants (*n* = 217, 62.2% female) were aged 45 to 90 years (*M* = 63.8, SD = 8.3) and were predominantly non-Hispanic whites (90.8%). Most were CU (92.2%) and had a parental history of dementia (73.3%). A large segment had high risk for cardiovascular disease (ASCVD >7.5, 35.2%) and genetic predisposition for dementia (*APOE*4+ = 33.2%). The asthma and control groups did not statistically differ on any of the sample characteristics. Asthma patients (*n* = 31) represented 14.2% of the sample, which is comparable to the prevalence of asthma (at 13.5%) among adults in the United States.^73^ The two groups were not statistically different in terms of PACC scores or CSF analyte concentrations (all *P* > 0.05, Table 2).

**Table 1:** Participant characteristics

Measure	Total	Control	Asthma
*N*	217	186	31
Age LP, m (SD)	63.83 (8.32)	63.56 (8.37)	65.49 (7.95)
ASCVD, m (SD)	8.84 (8.62)	8.41 (8.22)	11.19 (10.39)
Education, m (SD)	16.39 (2.33)	16.37 (2.31)	16.52 (2.5)
Female, *n* (%)	135 (62.2%)	118 (63.4%)	17 (54.8%)
Non-Hispanic White, *n* (%)	197 (90.8%)	171 (91.9%)	26 (83.9%)
Parental AD+, *n* (%)	159 (73.3%)	138 (74.2%)	21 (67.7%)
CU, *n* (%)	200 (92.2%)	170 (91.4%)	30 (96.8%)
Aβ+, *n* (%)	21 (10.5%)	19 (11.2%)	2 (6.7%)
Phospho-tau(181P)+, *n* (%)	12 (6.0%)	11 (6.5%)	1 (3.3%)
ASCVD+, *n* (%)	63 (35.2%)	49 (32.9%)	14 (46.7%)
*APOE*4+, *n* (%)	72 (33.2%)	63 (33.9%)	9 (29.0%)

Aβ, β-amyloid; Aβ+, Aβ42/Aβ40 < 0.046; AD + parental history of Alzheimer’s disease; *APOE*4 + apolipoprotein E ε4 carrier; ASCVD, atherosclerotic cardiovascular disease 10-year risk; ASCVD + ASCVD > 7.5; CU, cognitively unimpaired; LP, lumbar puncture; *M*, mean; *n*, number of observations; Phospho-tau(181P)+, Phospho-tau(181P)/Aβ42 > 0.038; *SD,* standard deviation.

**Table 2:** Participant cognition scores and CSF biomarker concentrations

Outcome	Control	Asthma	*P*-value
**Alzheimer’s disease pathology markers**			
Aβ42/Aβ40, m (SD)	0.066 (0.016)	0.065 (0.014)	0.677
Phospho-tau(181P) pg/mL, m (SD)	17.82 (7.55)	18.48 (9.21)	0.707
**Neurodegeneration markers**			
Neurogranin pg/mL, m (SD)	780.13 (328.82)	838.61 (426.35)	0.471
α-Synuclein pg/mL, m (SD)	161.74 (71.1)	161.75 (82.83)	0.999
NfL pg/mL, m (SD)	101.37 (86.21)	91.97 (45.63)	0.366
**Glial activation/neuroinflammation markers**			
YKL-40 ng/mL, m (SD)	147.2 (55.03)	152.24 (57.77)	0.653
sTREM2 ng/mL, m (SD)	8.22 (2.41)	8.21 (2.34)	0.977
GFAP ng/mL, m (SD)	9.47 (3.37)	8.77 (3.06)	0.254
S100B ng/mL, m (SD)	1.18 (0.27)	1.15 (0.19)	0.456
IL-6 pg/mL, m (SD)	4.98 (3.13)	4.98 (3.65)	0.994
**Cognitive Scores**			
PACC Baseline, m (SD)	0.462 (0.427)	0.418 (0.353)	0.539
PACC Slopes, m (SD)	-0.018 (0.017)	-0.016 (0.014)	0.462

Aβ, β-amyloid; GFAP, glial fibrillary acidic protein; IL-6, interleukin-6; *M*, mean; NfL, neurofilament light protein; PACC, preclinical Alzheimer cognitive composite; Phospho-tau(181P), Phosphorylated-tau-181; S100B, S100 calcium binding protein B; *SD*, standard deviation; sTREM2, soluble triggering receptor expressed on myeloid cells 2; YKL-40, chitinase-3-like protein 1.

### Whole brain TBSS and GBSS analysis summary

Whole brain TBSS analyses revealed that asthma strongly moderated the associations between white matter dMRI metrics and several CSF biomarkers, while overall group comparisons revealed no robust differences in brain microstructure in the omnibus test. Additionally, asthma moderated the relationships between dMRI metrics and age, PACC scores at baseline, as well as PACC slopes. Similarly, GBSS analyses revealed the moderating influence of asthma on the relationships between gray matter dMRI metrics and PACC variables, but this influence was less strong in relationships with age and CSF biomarkers. Overall, asthma was found to have an adverse moderating influence on brain microstructure. Table 3 summarizes the study findings on the influence of asthma on the associations between dMRI metrics and CSF biomarkers, age, and cognitive performance. See Supplementary information (sections 3–6 and Table 1) for all significant findings (corrected for multiple comparisons) within each dMRI metric, including those in which the omnibus test was not significant. Of note, asthma participants had greater white matter ODI than controls in the cerebral and cerebellar peduncles and the corticospinal tract; a finding that was consistent with and without additional covariates.

**Table 3:** Summary results of the influence of asthma

						Additional covariates	
Category	Predictor	Brain Region	Direction	Main model	ASCVD	*APOE*4	ASCVD & *APOE*4
Alzheimer’s disease pathology	Aβ42/Aβ40^a^	WM	↑	MD, RD	MD, RD	MD, RD	MD, RD
			↓	FA, NDI	FA, NDI	FA, NDI	FA, NDI
	Phospho-tau(181P)		↑	MD, RD, ODI	MD, RD, ODI	MD, RD, ODI	MD, RD, ODI
			↓	FA, NDI	FA, NDI	FA, NDI	FA, NDI
Synaptic integrity	Neurogranin	WM	↑	FISO, MD, ODI, RD	FISO, ODI	FISO, ODI	FISO, ODI
			↓	FA, NDI		FA	
Neuroinflammation/Glial activation	Log IL-6	WM	↑	FA, NDI	FA, NDI	FA, NDI	FA, NDI
			↓	AD, MD, ODI, RD	MD, RD	MD, RD	MD, RD
	S100B		↓	AD, FISO, MD, RD			
	YKL-40		↑			FISO	
Aging	Age	WM	↑	FISO, MD, RD			
			↓	FA			
Cognition	PACC slopes^a^	WM	↑	AD, MD, RD	RD*	RD*	RD*
			↓	FA, NDI	FA, NDI*	FA, NDI*	FA, NDI*
		GM	↑	AD, MD, RD			
			↓	NDI			
	PACC baseline^a^	WM	↓	FA			
		GM	↓	NDI	NDI	NDI	NDI

PALM analyses revealed moderating influence of asthma on associations between predictors and a range of dMRI metrics in both WM and GM, controlling for age, sex, and cognitive status. The models for age controlled for sex and cognitive status (cognitively unimpaired or not). PACC scores were z-scored with respect to data from all participants at the ADRC. PACC slopes were derived from change of PACC scores across assessment visits; analyses were re-run separately and jointly controlling for ASCVD and *APOE*4 to ascertain if the influence of asthma was independent or overlapping with the effects of these known risk factors of neurodegeneration. All findings unless otherwise indicated were FWE-corrected *P* < 0.05 for the specified dMRI metric, and for the omnibus test across all dMRI metrics. Arrows in the ‘Direction’ column indicate direction of the moderating influence of asthma for the dMRI metrics listed for each model. AD, axial diffusivity; ADRC, Wisconsin Alzheimer’s Disease Research Center; Aβ, β-amyloid; *APOE*4, apolipoprotein E ε4 carrier; ASCVD, atherosclerotic cardiovascular disease 10-year risk; dMRI, diffusion MRI; FA, fractional anisotropy; FISO, isotropic volume fraction; FWE, familywise error; GM, gray matter; IL-6, interleukin-6; MD, mean diffusivity; NDI, neurite density index; ODI, orientation dispersion index; PACC, preclinical Alzheimer cognitive composite; PALM, permutation analysis of linear models; Phospho-tau(181P), Phosphorylated-tau-181; RD, radial diffusivity; S100B, S100 calcium binding protein B; WM, white matter; YKL-40, chitinase-3-like protein 1. ^a^Direction of change for dMRI metrices for Aβ42/Aβ40, and PACC variables are reversed for easier understanding, as lower values indicate more adverse impact.

* Omnibus FWE corrected *P* < 0.1.

### Asthma moderates the relationship between dMRI metrics and CSF biomarkers

**Figure 2:**
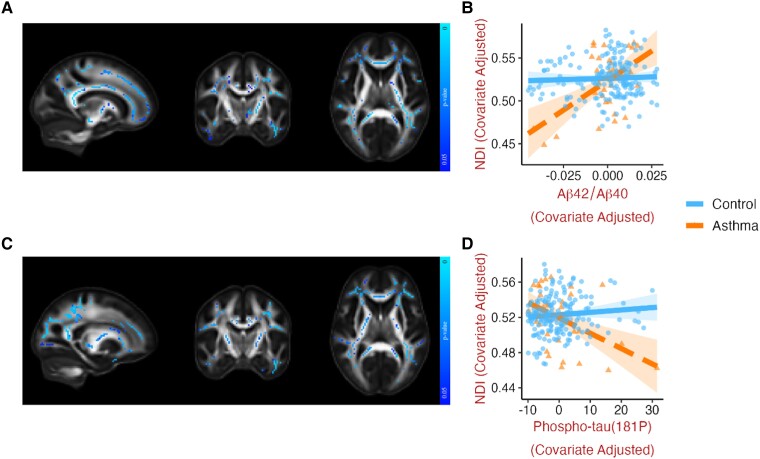
**Asthma moderates the relationship between Alzheimer’s disease pathology biomarkers and dMRI metrics.** Representative slices of white matter template displaying voxels where asthma significantly moderated (at *P* < 0.05, FWE corrected) the relationships between (**A**) Aβ42/Aβ40 and NDI (median β = 0.238, *t* = 3.947, *P* < 0.001), and (**C**) Phospho-tau(181P) and NDI (median β = -0.245, *t* = -3.872, *P <* 0.001). Corresponding scatterplots (**B** and **D**) generated for visualization by extracting the mean of all significant voxels, with one data point for each individual, adjusted for age, sex and cognitive status and removing one influential outlier. Note that lower Aβ42/Aβ40 and higher Phospho-tau(181P) values are indicative of higher pathology. The moderating influence of asthma was also found for the associations between Aβ42/Aβ40 and FA, MD, and RD (Supplementary Fig. 1A–C), and Phospho-tau(181P) with FA and MD (Supplementary Fig. 2A–D). All relationships remained significant when controlling for ASCVD and/or *APOE*4 genotype. Brain images are shown in radiological convention (left hemisphere is shown on the right side on coronal and axial views). Aβ42/Aβ40 ratio, β-amyloid 42/β-amyloid 40; APOE4, apolipoprotein **E** ε4 allele carrier; ASCVD, atherosclerotic cardiovascular disease; dMRI, diffusion MRI; FA, fractional anisotropy; FWE, familywise error; MD, mean diffusivity; NDI, neurite density index; Phospho-tau(181P), phosphorylated-tau-181; RD, radial diffusivity.

Lower Aβ42/Aβ40 was associated with lower NDI (Fig. 2A and B) and FA, as well as higher MD and RD (Supplementary Fig. 1A–C) in asthma relative to controls. Similarly, the anticipated relationships of higher values of Phospho-tau(181P) with lower NDI (Fig. 2C and D), lower FA, and higher MD, RD and ODI (Supplementary Fig. 2A­D) were more pronounced in asthma patients. Both these effects were widespread in white matter and remained consistent after individually and jointly controlling for ASCVD and *APOE*4. Analogous moderating effects of asthma were not observed in gray matter.

**Figure 3:**
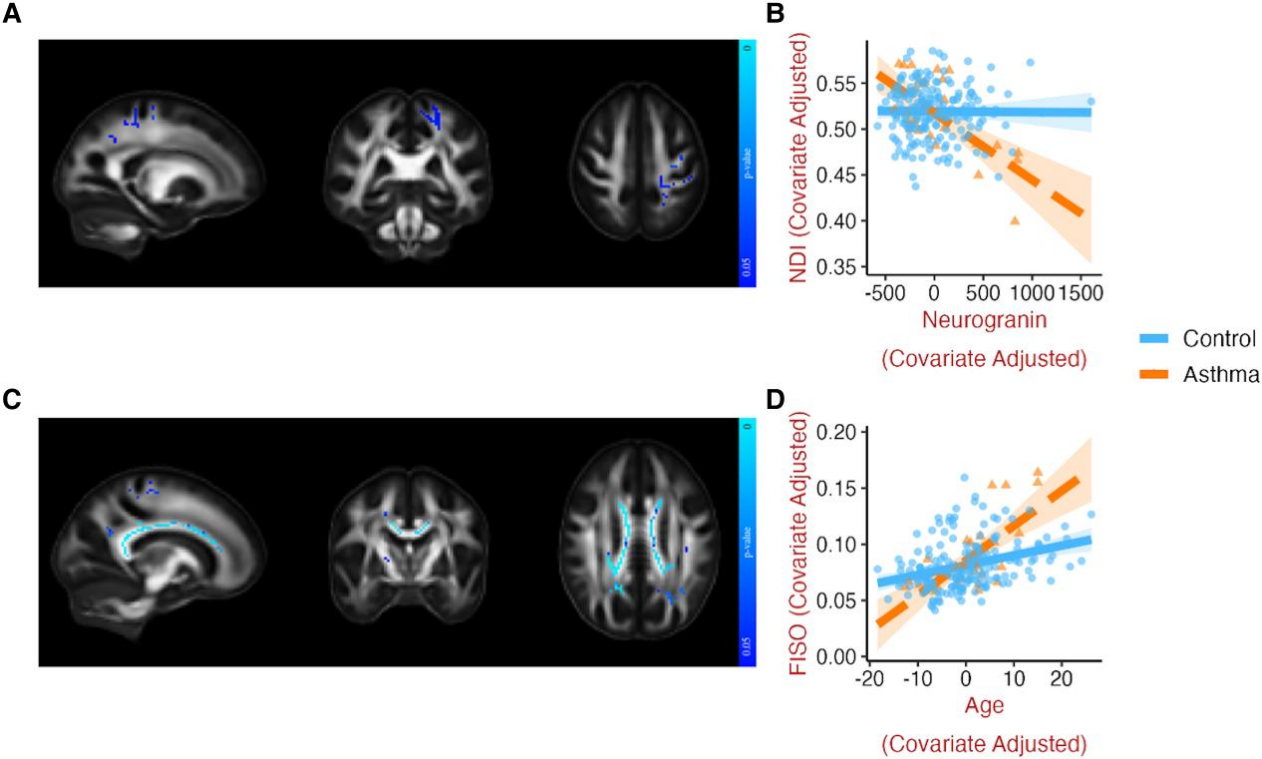
**Asthma moderates the relationships between dMRI metrics, CSF neurogranin, and age.** Representative slices of white matter template displaying voxels where asthma significantly moderated (at *P* < 0.05, FWE corrected) the relationships between (**A**) neurogranin and NDI (median β = -0.311, *t* = -4.493, *P* < 0.001), and (**C**) age and FISO (median β = 0.242, *t* = 3.77, *P* < 0.001). Corresponding scatterplots (**B** and **D**) generated for visualization by extracting the mean of all significant voxels, with one data point for each individual, adjusted for sex and cognitive status (and age, in the case of neurogranin). The influence of asthma was also found for associations between neurogranin and FA, MD, RD, ODI, and FISO (Supplementary Fig. 3A–E) and between age and FA, MD, and RD (Supplementary Fig. 6A–C). Brain images are shown in radiological convention (left hemisphere is shown on the right side on coronal and axial views). dMRI, diffusion MRI; FA, fractional anisotropy; FISO, isotropic volume fraction; FWE, familywise error; MD, mean diffusivity; NDI, neurite density index; ODI, orientation dispersion index; RD, radial diffusivity.

Negative relationships between neurogranin concentrations and FA and NDI, and positive relationships between neurogranin and MD, RD, ODI, and FISO were present in the asthma group, but not among controls. Whereas these effects were bilateral and widespread for FA and RD, they were more localized for the other metrics (Supplementary Fig. 3A–E). For example, for NDI (Fig. 3A and B), a stronger negative association with neurogranin in those with asthma was found in the corticospinal tract, superior longitudinal fasciculus, anterior corona radiata and uncinate fasciculus in the left hemisphere. When additional covariates were included in these models, most, but not all, of the effects persisted (Table 3). Specifically, the stronger negative association of neurogranin with FA in those with asthma was consistent when controlling for *APOE*4 but not when ASCVD was included as a covariate, whereas the stronger positive associations of neurogranin with FISO and ODI were consistent across models that included ASCVD and *APOE*4. See supplementary information (section 3) for additional findings related to neurogranin and α-synuclein in gray matter, where the omnibus test was not significant.

**Figure 4:**
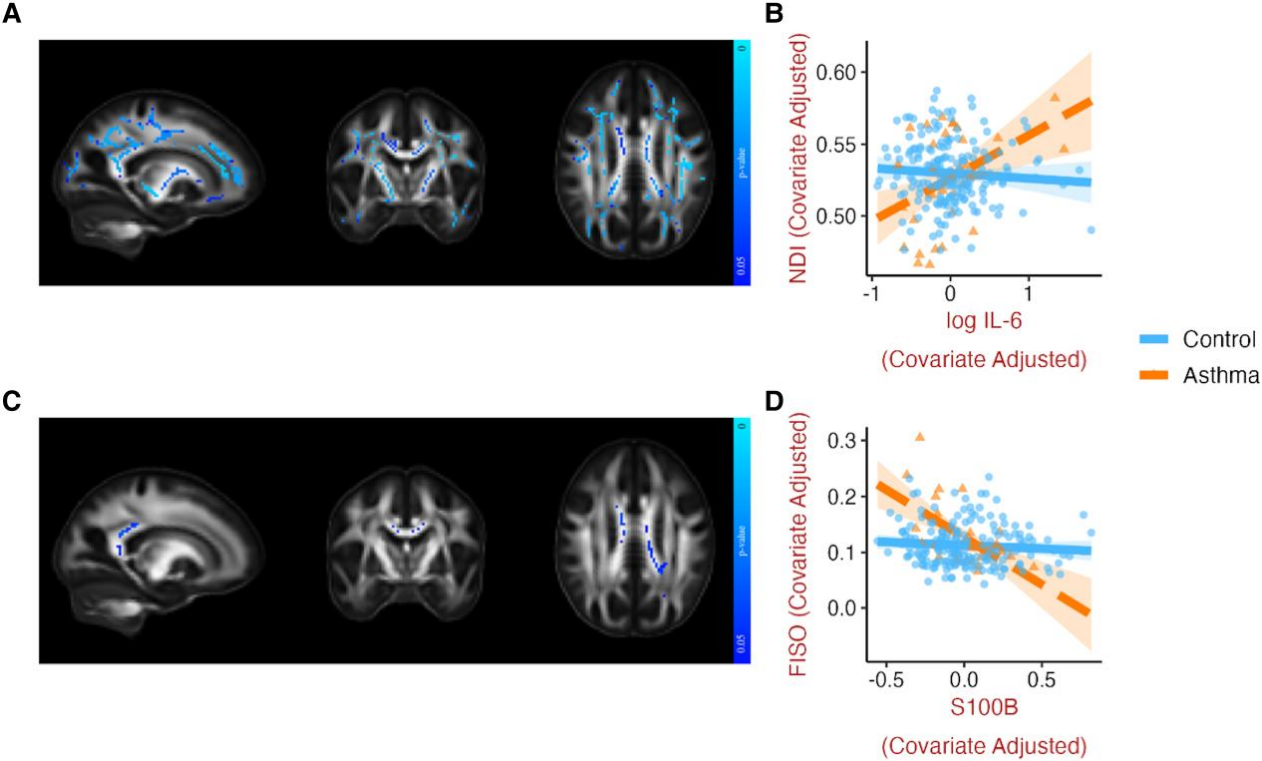
**Asthma moderates the relationships between dMRI metrics and CSF biomarkers of neuroinflammation (IL-6) and glial activation (S100B).** Representative slices of white matter template displaying voxels where asthma significantly moderated (at *P* < 0.05, FWE corrected) the relationships between (**A**) log IL-6 and NDI (median β = 0.209, *t* = 3.357, *P* = 0.001), and (**C**) S100B and FISO (median β = -0.243, *t* = -4.001, *P* < 0.001). Corresponding scatterplots (**B** and **D**) generated for visualization by extracting the mean of all significant voxels, with one data point for each individual, adjusted for age, sex, and cognitive status. The influence of asthma was also found for associations between log IL-6 and FA, MD, RD, AD and ODI (Supplementary Fig. 4A–E), as well as between S100B and MD, RD, and AD (Supplementary Fig. 5A–C). Brain images are shown in radiological convention (left hemisphere is shown on the right side on coronal and axial views). AD, axial diffusivity; CSF, cerebrospinal fluid; dMRI, diffusion MRI; FA, fractional anisotropy; FISO, isotropic volume fraction; FWE, familywise error; IL-6, interleukin-6; MD, mean diffusivity; NDI, neurite density index; ODI, orientation dispersion index; RD, radial diffusivity; S100B = S100 calcium binding protein B.

There were unexpected relationships between IL-6 and S100B and white matter microstructure in those with asthma, but not controls. In white matter, asthma moderated the influence of IL-6 on dMRI metrics such that in asthma, higher IL-6 concentrations were associated with higher values of NDI (Fig. 4A and B) and FA, as well as lower MD, RD, AD, ODI (Supplementary Fig. 4A–E), whereas this relationship was absent in the control group. These effects were widespread and, except for AD and ODI, consistent with and without additional covariates. Similarly, in asthma but not controls, higher concentrations of the glial marker S100B were associated with lower FISO (Fig. 4C and D), MD, RD, and AD (Supplementary Fig. 5A–C) in the corpus callosum and parts of the corona radiata. However, these effects were no longer present when adjusting for cardiovascular or genetic risk. We did not find a significant influence of asthma on the relationships between IL-6 or S100B and dMRI metrics in gray matter. See supplementary information (section 4) for additional findings, where the omnibus test was not significant, showing an adverse influence of asthma on the relationship between other biomarkers of glial activation, YKL-40 and sTREM2, and white and gray matter microstructure.

### Asthma amplifies the relationships between dMRI metrics, age, and cognitive decline

Deterioration in white matter microstructure with advancing age, reflected in higher FISO (Fig. 3C and D), lower FA, and higher MD and RD (Supplementary Fig. 6A–C), was amplified in asthma relative to controls, in the corpus callosum, posterior corona radiata, and internal capsule. See supplementary information (section 5) for additional findings, where the omnibus test was not significant, related to the influence of asthma on the deleterious effects of age in white and gray matter.

**Figure 5:**
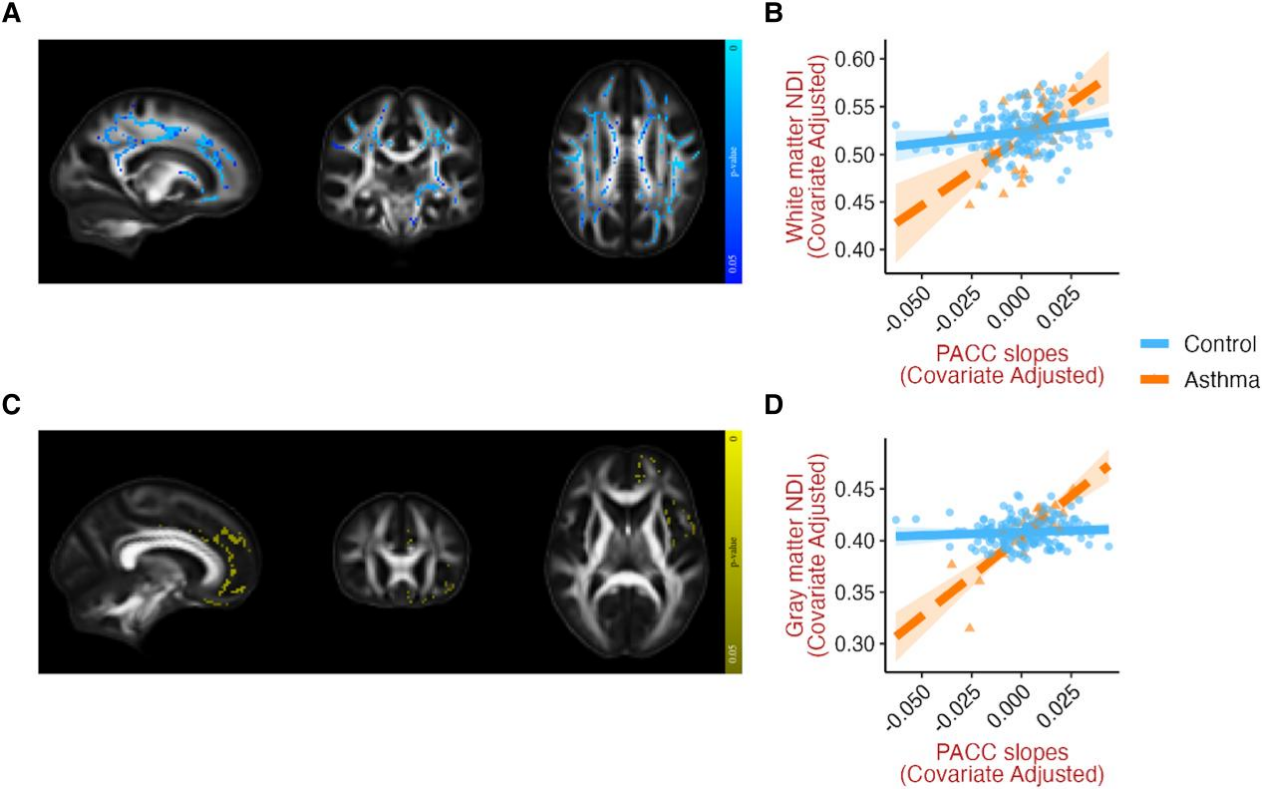
**Asthma moderates the relationship between dMRI metrics in white and gray matter and PACC slopes.** Representative slices of white and gray matter templates displaying voxels where asthma significantly moderated (at *P* < 0.05, FWE corrected) the relationships between PACC slopes and NDI in (**A**) white matter (median β = 0.221, *t* = 3.799, *P* < 0.001), and (**C**) gray matter (median β = 0.334, *t* = 5.95, *P* < 0.001). Corresponding scatterplots (**B** and **D**) generated for visualization by extracting the mean of all significant voxels, with one data point for each individual, adjusted for age, sex, cognitive status, and PACC scores at baseline. Similar influence of asthma was found on the associations between FA, MD, AD, and RD with PACC slopes (Supplementary Fig. 7A–D). Brain images are shown in radiological convention (left hemisphere is shown on the right side on coronal and axial views). AD, axial diffusivity; dMRI, diffusion MRI; FA, fractional anisotropy; FWE, familywise error; MD, mean diffusivity; NDI, neurite density index; PACC, preclinical Alzheimer cognitive composite; RD, radial diffusivity.

Asthma was also associated with a stronger relationship between dMRI metrics and PACC slopes in both white and gray matter. Relative to controls, lower NDI (Fig. 5A and B) and FA, and higher MD, RD, and AD (Supplementary Fig. 7A–D) in white matter were associated with more negative PACC slopes in asthma, indicative of accelerated cognitive decline over time. These results were consistent with and without additional covariates. At baseline, lower PACC scores were associated with lower white matter FA (Supplementary Fig. 8A and B) in asthma relative to controls, but this effect did not persist when adjusting for the additional covariates. In gray matter, lower NDI (Fig. 5C and D) and higher MD, RD, and AD were associated with steeper negative PACC slopes in those with asthma relative to controls, in areas that include the medial frontal cortex, precentral gyrus, and temporal pole. However, these effects were not found when ASCVD and *APOE*4 were included as covariates. At baseline, lower PACC scores were associated with lower gray matter NDI in the right supramarginal gyrus, in asthma relative to controls (Supplementary Fig. 8C and D), and this effect remained consistent after adjusting for the additional covariates.

## Discussion

Relationships between diffusion-weighted MRI indices of neurodegeneration, CSF biomarkers, and decline in cognitive function were consistently stronger in those with asthma, providing evidence that, relative to the control group, in asthma, brain microstructural changes detectable in predominantly CU volunteers are more strongly associated with neuropathology, including that related to Alzheimer’s disease. This implies that for the same degree of dementia-related pathology, those with asthma have more adverse white- and gray-matter microstructural changes, and poorer prognosis in terms of disease progression. We previously reported widespread white matter microstructural differences between asthma and controls.^24^ Our current results, utilizing an older sample, extend the biological significance of these findings by anchoring their interpretation to associations with established CSF biomarkers of neuropathology, while providing information related to the regional specificity and extent of structural changes. Most of our findings remained consistent after controlling for cardiovascular and genetic risk factors, suggesting that the deleterious effects of asthma on brain health are at least partially independent of these risk factors.

We examined white and gray matter microstructural properties in asthma using dMRI metrics that provide complementary information. For example, NDI measures diffusivity within axons and is largely indicative of axonal integrity, primarily myelination, in white and gray matter.^28^ Reductions in NDI are therefore suggestive of degenerative changes including demyelination and/or neuronal loss.^48^ On the other hand, ODI represents neurite dispersion and reflects the spread of neurites in gray and white matter. Thus, higher white matter ODI reflects axonal disorganization.^49^ See supplementary information section 7 ‘Interpretation of dMRI metrics’ for an overview on how dMRI metrics can help detect alterations in white and gray matter microstructure that are pertinent to the present study. Together, these metrics allow interpretation about the nature of the moderating influence of asthma on the degenerative effects of sub-clinical pathology seen in our sample.

In our predominantly CU sample, individuals with asthma had consistently stronger, robust, and widespread relationships between amyloid pathology and white matter NDI, FA, MD, and RD, suggesting that reduced axonal integrity and myelination are related to AD-specific pathology in asthma. Definitive diagnosis of Alzheimer’s disease is not achieved until autopsy, yet its core biomarkers, amyloid-β and phosphorylated tau, have high diagnostic and prognostic value prior to the onset of dementia^5^ and have been previously associated with early brain microstructural changes in CU participants.^74^ It is important to note that we did not find overall group differences in amyloid or tau pathology, or for that matter, in any of the dMRI measures and CSF biomarkers assessed in our study. Thus, the strong moderating influence of asthma on the relationships between multiple measures of brain health assessed in our study demonstrates that asthma accelerates neurodegenerative processes well before diagnosis is possible using established biomarkers. Previous reports suggest that among CU, those who are amyloid-positive show steeper reduction in cortical NDI associated with increasing phosphorylated tau; in turn, those who are tau-positive, show steeper NDI reduction with lower Aβ42/Aβ40.^74^ It is noteworthy that this pattern of NDI reduction with greater tau and amyloid pathology was observed, despite the lack of amyloid- or tau-positivity, among those with asthma in our study.

Consistent with these findings of early NDI reduction that are suggestive of impaired myelination among CU on the Alzheimer’s disease continuum, single-nucleus RNA sequencing has shown differential expression of genes related to regulation of myelination in early Alzheimer’s disease pathology.^75^ Among those with early-onset Alzheimer’s disease, reduced NDI has been found across the gray matter and superficial white matter, suggestive of loss of myelinated fibres.^76^ In our study, asthma appears to contribute to, and augment, the reduced axonal integrity and myelination seen with increasing Alzheimer’s disease pathology, independent of cardiovascular and genetic risk factors. It is possible that asthma also acts synergistically with the faster loss of myelination seen among *APOE*4 carriers.^77^ In cases of mild cognitive impairment and in early stages of Alzheimer’s disease, white matter microstructural changes have been consistently observed in the corpus callosum and cingulum bundles,^48^ as well as in the fornix, uncinate fasciculus and precuneus.^78^ The strong spatial overlap of our findings with areas consistently impacted by Alzheimer’s pathology, and relationships with Alzheimer’s disease biomarkers Aβ42/Aβ40, and Phospho-tau(181P), indicates that for those who are already on the Alzheimer’s disease continuum, asthma is likely to speed up the progression to dementia.

The influence of asthma on the relationships between brain microstructure and CSF biomarkers of synaptic degeneration, as well as with cognitive decline, is noteworthy. Synaptic degeneration is considered to be a strong correlate of cognitive deficits^79^ that are an early substrate of Alzheimer’s disease.^80^ CSF neurogranin is a biomarker for synaptic degeneration and higher concentrations have been found in those with mild cognitive impairment and Alzheimer’s disease.^81,82^ CSF neurogranin has been shown to offer prognostic value among CU and has been diagnostic among early symptomatic Alzheimer’s disease.^83^ In our study, the stronger relationships of neurogranin with NDI, RD, and FA in asthma overlapped spatially in the left corticospinal tract and left superior longitudinal fasciculus suggesting that in these regions, myelination was compromised. Further, the spatial overlap of these effects in white matter FA and ODI in the forceps minor, uncinate fasciculus, anterior corona radiata, and the genu of the corpus callosum is suggestive of axonal disorganization in asthma.^84^ In line with our findings, an inverse relationship between CSF neurogranin and white matter FA has been previously reported in a longitudinal study of CU individuals.^85^ We have previously reported that in severe asthma, neurogranin concentrations are elevated relative to controls.^25^ Although sample size constraints preclude analysis by asthma severity in the present study, here we found evidence of steeper cognitive decline associated with lower NDI and higher MD and RD that were spread across white matter and frontal gray matter, in those with asthma relative to controls. Taken together, our findings suggest that axonal disorganization and compromised myelination may contribute to, and have an out-sized impact on, cognitive decline in asthma.

Older age is a principal risk factor for dementia,^80^ due to accumulation of multiple pathologies^86^ and ‘inflammaging’, the progressive increase in chronic sub-clinical inflammation with age.^87–89^ White matter microstructure across a number of tracts has been found to be sensitive to aging with measurable changes in diffusion imaging metrics.^90^ Among older CU individuals, white matter microstructural differences (lower NDI and higher MD) in the cingulum have been shown to partially mediate the inverse association between age and executive function.^48^ Overall, among CU adults, increasing age is associated with maturational, followed by degenerative alterations to white matter microstructure^91^ and in our older study sample, these age-related degenerative changes were amplified in asthma. We did not have access to details related to duration of asthma in our participants, so it is not possible to definitively determine if the accelerated aging effects we see are unrelated to disease duration. Nevertheless, asthma is a chronic condition that often begins in childhood and, given the age range of participants in our sample, it is likely that most of the participants with asthma have lived with the disease for several decades. Individuals with other co-morbid chronic inflammatory conditions were excluded from our study and all analyses covaried for age, so our findings demonstrate the pronounced influence of asthma over and above the effect of normal aging.

Our findings related to the influence of asthma on the relationship between brain microstructure and CSF concentrations of S100B and IL-6 suggest a complex immune profile in those with asthma. S100B is abundantly found in astrocytes, is elevated in the CSF of those with Alzheimer’s disease and is considered a marker of acute neural distress that is neuroprotective at nanomolar concentrations and neurotoxic at micromolar levels.^55,92^ In our study, CSF S100B concentrations were in the nanomolar range, and although these are not tissue concentrations, they are suggestive of neurotrophic astroglial activity. Similarly, the consistent and widespread relationship between CSF IL-6 and dMRI indicators of better brain health in those with asthma was surprising. Peripheral IL-6 is elevated in Alzheimer’s disease, although this relationship is not consistently seen with CSF IL-6.^93,94^ Peripheral IL-6 is also elevated in asthma and is thought to contribute to the pathophysiology of at least one asthma endotype.^56,95^ However, IL-6 is a pleiotropic cytokine that switches its role from neuroprotective under normal physiological conditions to neurotoxic at higher concentrations following acute CNS injury and in chronic CNS diseases.^96–98^ In our study, CSF IL-6 levels were within the normal range.^97^ Another consideration is the temporal pattern of biomarker expression on the Alzheimer’s disease continuum.^5^ It is possible that the seemingly salubrious associations of CSF IL-6 and S100B with brain microstructure in asthma may reverse in participants farther along the progression to dementia, and merits longitudinal assessment. Further, in contrast to the counterintuitive effects seen with IL-6 and S100B, it is noteworthy that we also found some evidence that asthma moderated associations that appear to be deleterious between glial activity (indicated by YKL-40 and sTREM2) and both white and gray matter microstructure (see Supplementary material (section 4)). Taken together, our findings point to a complex interplay among multiple CNS immune mediators and brain microstructure.

In our study, non-asthma controls showed expected deleterious effects of age on brain microstructure^48,78^ that are associated with cognitive decline.^99^ Further, relationships between CSF biomarkers and brain microstructure were weak or absent among controls, in line with previous reports among amyloid and tau negative CU individuals.^74^ Thus, the moderating effects of asthma seen in our study suggest that asthma exacerbates the effects of aging and emerging pathology and accelerates the rate of pre-clinical cognitive decline before clinical symptoms are detected. These findings add to the body of literature indicating that systemic inflammation can contribute to and augment progression to dementia^2,100^ and that the ensuing complex and multiphasic glial activity can have both protective and adverse effects that appear to arise independently of, in addition to being reactive to, the aggregation of amyloid into soluble oligomers and plaques as per the amyloid cascade hypothesis.^26,100–103^

We evaluated both DTI and NODDI estimates, on a voxelwise basis, to determine white and gray matter microstructural properties in asthma. In general, we found high concordance across dMRI metrics with several CSF biomarkers of neuropathology in asthma, suggesting that these imaging techniques provide a sensitive and non-invasive way to track pre-clinical changes in neuropathological markers, in the context of systemic inflammation. We applied the NODDI model to our data, which fits a biophysical, multi-compartment diffusion-model to each voxel, so that the derived metrics (NDI, ODI and FISO) offer more biologically interpretable findings.^29^ NODDI has been validated with histology and ex-vivo DWI^84^ and the derived maps have regional specificity^104^ and sensitivity to detect intra-individual temporal changes.^28^ Our robust findings of stronger associations between CSF biomarkers and NDI, ODI and FISO in asthma patients as compared to controls add nuance to the evidence for the microstructural sensitivity of NODDI.

Although our study advances our understanding of the biological meaning of previous neuroimaging findings in asthma and informs the regionality and extent of neuropathological changes indicated by CSF biomarkers, we do not yet have a mechanistic understanding of how asthma contributes deleteriously to brain health. There is a rapidly growing literature on brain-immune interactions establishing how systemic inflammation leads to increased blood-brain barrier permeability^1^ and immune activity and dysregulation in the CNS.^105^ There is also mounting evidence of neuroimmune communication along the lung-brain axis suggesting that airway inflammation due to pathogen exposure and sterile lung injuries can contribute to neuroinflammation.^10,106^ Airway inflammation is known to provoke neuroinflammation in animal models of asthma,^9^ potentially via circumventricular organs,^17,106^ and there is increasing evidence suggesting that neuroinflammation contributes to neurodegeneration and Alzheimer’s disease pathophysiology.^101,103,107^ In addition to direct effects of airway inflammation on the brain, there are multiple pathways through which asthma may influence brain health. For example, asthma is known to affect the vasculature^108,109^ and vascular dysfunction contributes to Alzheimer’s disease etiopathogenesis.^110,111^ Alternatively, hypoxia could mediate the influence of asthma on brain health.^33,112^ In reality, a combination of mechanisms likely give rise to the relationships reported here. The present study begins to elucidate the pathology that underlies early brain microstructural changes and cognitive decline that may progress to the increased prevalence of dementia in asthma reported in the literature.^18–20,113,114^

The present study contributes to the growing literature on neuroimmune interactions, emphasizing the importance of minimizing systemic inflammation for preserving brain health and preventing or delaying dementia onset in affected individuals.^2^ We extend our recent findings of deleterious effects of asthma on the brain by anchoring brain imaging evidence in validated CSF measures of neuropathology and longitudinal comprehensive cognitive assessments.^24^ There were noteworthy differences between these two studies that require comment. Firstly, participants in our previous study^24^ were younger, and thus much less likely to show age-related decline in brain health. The early and protracted impact of asthma on brain microstructure among cognitively intact individuals was therefore likely to be more stark when compared to age-matched controls. On the other hand, the present study sample was older and enriched for parental history of Alzheimer’s disease. The impact of asthma on brain microstructure was therefore compared to a control group more likely to have deteriorating brain health, leading to less prominent group differences. Secondly, there were important sample size differences (*n* = 111 asthma versus 135 controls in the previous study; *n* = 31 asthma versus 186 controls in the present study) limiting our ability to detect the effect of asthma over and above the ongoing brain changes unrelated to asthma in these individuals. Our study has several strengths. We leveraged several recent developments: a comprehensive panel of CSF biomarkers relevant for Alzheimer’s disease,^32^ a preclinical cognitive composite that is sensitive to early signs of decline^57,58^ and the NODDI model applied to multi-shell diffusion-weighted imaging.^29^ In addition, our approach was highly rigorous; our analyses were constrained to conservative gray and white matter masks that included only voxels with high representation across participants^53^ and we examined effects of asthma while controlling for other established risk factors. Finally, our study sample was predominantly CU but enriched for parental history of Alzheimer’s disease, providing a rare dataset to examine the amplifying influence of asthma in vulnerable individuals in the absence of frank dementia.

Nevertheless, there are several important limitations that need to be considered. Although we limited our analyses to carefully determined skeletonized masks of gray and white matter, given the large voxel sizes relative to cortical thickness, especially in an aging population, we cannot rule out the possibility of some partial volume effects. Additionally, even though the overall study sample is large, and the proportion of asthma participants is as expected,^73^ the sample of 31 asthma participants is relatively small. As such we were unable to examine the influence of asthma severity or examine sub-groups characterized by amyloid and tau positivity to ascertain impact on those further along the Alzheimer’s disease continuum.^6^ As this was a retrospective study, we were dependent on prescription medication to determine asthma status. Future prospective studies focused on dementia would benefit from physician diagnosis of asthma, as well as other chronic inflammatory diseases, and documentation of disease exacerbations and medication usage.

In summary, our study provides multi-modal, convergent evidence that asthma is associated with indicators of neurodegeneration and poorer brain health including pathology specific to Alzheimer’s disease, that in turn is associated with accelerated cognitive decline over time. Currently, uncontrolled asthma is a significant public health challenge, despite the availability of a range of effective treatments.^11,115^ Considering the high prevalence of asthma, the racial and economic disparities in asthma morbidity,^116^ and the lifelong nature of this disease, concerted efforts toward improvement in asthma management,^117,118^ as well as identification and targeting of key signaling pathways involved in the relationships reported here would improve both lung and brain health and has the potential to make a meaningful impact on the public health burden and societal costs associated with dementia.

## Supplementary Material

fcad180_Supplementary_Data

## Data Availability

All primary data used in the study are available to approved investigators. Further information is available at https://www.adrc.wisc.edu/research-services-and-resources.

## Abbreviations

Aβ =: β-amyloid
AD =: axial diffusivity
ADRC =: Wisconsin Alzheimer’s Disease Research Center
ANTs =: advanced normalization tools
*APOE4+* =: apolipoprotein E ε4 allele carrier
ASCVD =: atherosclerotic cardiovascular disease
BRAVO =: brain volume imaging sequence
CU =: cognitively unimpaired
DIPY =: diffusion imaging in python
DMIPY =: diffusion microstructure imaging in python
dMRI =: diffusion MRI
DTI =: diffusion tensor imaging
DWI =: diffusion weighted imaging
FA =: fractional anisotropy
FMRIB =: Functional MRI of the Brain
FSL =: FMRIB software library
FWE =: familywise error
FISO =: isotropic volume fraction
GBSS =: gray-matter based spatial statistics
GFAP =: glial fibrillary acidic protein
GM =: gray matter
IL-6 =: interleukin-6
LP =: lumbar puncture
*M* =: mean
MD =: mean diffusivity
NDI =: neurite density index
NfL =: neurofilament light
NIH =: National Institutes of Health
NODDI =: neurite orientation dispersion and density imaging
NPC =: non-parametric combination
NTK =: NeuroToolKit
ODI =: orientation dispersion index
PACC =: preclinical Alzheimer cognitive composite
PALM =: permutation analysis of linear models
Phospho-tau(181P) =: phosphorylated-tau-181
RD =: radial diffusivity
S100B =: S100 calcium binding protein B
SD =: standard deviation
sTREM2 =: soluble triggering receptor expressed on myeloid cells 2
TBSS =: tract-based spatial statistics
TE =: echo time
TR =: repetition time
WM =: white matter
YKL-40 =: chitinase-3-like protein 1
