# Supplementary material for: Impact of asthma on the brain: evidence from diffusion MRI, CSF biomarkers and cognitive decline
Source: Brain Commun. 2023 Jun 13;5(3):fcad180. doi: 10.1093/braincomms/fcad180 (PMC10292933; doi:10.1093/braincomms/fcad180)
Supplement: fcad180_Supplementary_Data [file fcad180_supplementary_data.docx]

# Supplementary material:

Impact of asthma on the brain: evidence from diffusion MRI and CSF biomarkers and cognitive decline

Ajay Kumar Nair,^1^ Carol A. Van Hulle,^2,3^ Barbara B. Bendlin,^2–4^ Henrik Zetterberg, ^5–9^ Kaj Blennow,^5,6^ Norbert Wild,^10^ Gwendlyn Kollmorgen,^10^ Ivonne Suridjan,^11^ William W. Busse,^3^ Douglas C. Dean III,^12–14^ and Melissa A. Rosenkranz ^1,15^

# Supplementary material

## Additional exclusion criteria based on comorbidities

Participants with the following CNS comorbid conditions were excluded: stroke, Parkinson’s disease, transient ischemic attack, essential tremor, hydrocephalus, meningioma, brain or cerebellar aneurysm, epilepsy, seizures, subarachnoid hemorrhage, ataxia, progressive supranuclear palsy, and coma. Additionally, all non-asthma participants with the following immune comorbidities were excluded: rheumatoid arthritis, psoriasis, inflammatory bowel disease, Crohn’s disease, ulcerative colitis, irritable bowel syndrome, systemic lupus erythematosus, multiple sclerosis, autoimmune cholangitis, and polymyalgia rheumatica.

## Asthma medications

The following medications were used to determine asthma status: inhaled steroids (beclomethasone, budesonide, fluticasone, and mometasone); systemic steroids when taken in conjunction with other asthma medications (prednisone, methylprednisolone, betamethasone, dexamethasone, and hydrocortisone); biologics with other asthma medications (omalizumab); long-acting beta-agonists combined with inhaled steroids (budesonide-formoterol, fluticasone-salmeterol, fluticasone-vilanterol, and formoterol-mometasone); leukotriene antagonists (montelukast); and bronchodilators (albuterol, albuterol-ipratropium, ipratropium, theophylline, formoterol, salmeterol, and olodaterol-tiotropium).

## Additional results on the relationship between dMRI metrics and CSF biomarkers of synaptic degeneration

There were tentative findings with biomarkers of neurodegeneration (Supplementary Table 1), where the omnibus test was not significant. In cerebellar gray matter, relative to controls, the asthma group showed a stronger positive relationship between FA and neurogranin and a stronger negative relationship between ODI and neurogranin—both moderating effects were consistent with and without inclusion of additional covariates. Asthma also moderated the relationship between a second synaptic degeneration biomarker, α-synuclein, and dMRI metrics such that stronger positive associations between α-synuclein and white matter ODI in the right anterior corona radiata, splenium of corpus callosum, forceps minor and forceps major as well as cerebellar gray matter FA were found for those with asthma relative to controls. The latter effect was consistent with and without inclusion of additional covariates.

## Additional results on the relationship between dMRI metrics and CSF biomarkers of glial activation and neuroinflammation

Adverse moderating effects of asthma were found on relationships between other glial biomarkers and dMRI metrics (Supplementary Table 1), where the overall omnibus test was not significant. In the model additionally controlling for *APOE*4 (but not in models that lacked this covariate), asthma patients had increased white matter FISO in the left uncinate fasciculus and left inferior fronto-occipital fasciculus with increasing YKL-40, an effect that was not seen among controls. An analogous effect was observed in gray matter in the temporal fusiform cortex and parahippocampal gyrus, in models controlling for ASCVD and/or *APOE*4. Further, in asthma, but not in controls, there was a positive relationship between sTREM2 and right cerebellar gray matter FA in the main model, as well as when controlling for ASCVD or *APOE*4 but not when both ASCVD and *APOE*4 were included in the model.

## Additional results on the age-related impact of asthma on dMRI metrics

The amplifying effect of asthma on the positive relationship between age and white matter FISO was consistent when individually and jointly controlling for ASCVD and *APOE*4, but the omnibus test across modalities was not significant in the models with the additional covariates.

There were analogous moderating effects of asthma on the association between age and gray matter microstructure that were less pervasive across dMRI metrics than those seen in white matter. Relative to controls, the increase in MD, AD, and RD in the right temporal pole with increasing age was more pronounced in asthma. In this analysis, the omnibus test across metrics was at a trend level of significance (*P <* 0.1, corrected) and this effect was not seen in models controlling for ASCVD or *APOE*4. On the other hand, in models controlling for *APOE*4, the positive association between age and FISO in gray matter areas, including the angular gyrus, middle and inferior temporal gyri and the parahippocampal gyrus, was more pronounced in asthma.

## Asthma moderates the relationship between white and gray matter dMRI metrics with baseline cognition

Asthma accelerated the relationship between DWI metrics and baseline PACC scores such that relative to controls, asthma patients with lower baseline PACC scores had lower FA in white matter (Supplementary Fig. 8A and 8B) and lower NDI in gray matter (Supplementary Fig. 8C and 8D). When additionally controlling for ASCVD and/or *APOE*4, asthma patients with lower baseline PACC scores had higher MD, RD, and AD in both white and gray matter (Table 3).

## Interpretation of dMRI metrics

The various dMRI metrics provide complementary, and at times overlapping, information on tissue microstructure that is brain region dependent. Together, these metrics allow interpretation about the nature of the moderating influence of asthma on the degenerative effects of sub-clinical pathology seen in our study. NDI measures diffusivity within axons and is largely indicative of axonal integrity, primarily myelination, in WM and GM.^1^ Reductions in NDI are therefore suggestive of degenerative changes including demyelination or neuronal loss.^2^ ODI represents neurite dispersion and reflects spread of dendrites in GM and axons in WM. In GM, lower ODI reflects loss of dendritic arborization, whereas in white matter, higher values of ODI reflects axonal disorganization,^3^ but has also been shown to be acutely induced by changes in microglial density.^4^ FISO measures isotropic diffusion^5^ and thus higher FISO values could be the result of increased free water content due to reduction in neurite density or orientation, and may also be influenced by changes in glial activity and/or morphology. The DTI metrics (FA, MD, RD, and AD) are not based on biophysical models and are sensitive to changes that are more difficult to directly interpret on their own, as they are affected by many factors such as axonal number, extent of myelination and membrane permeability, orientational dispersion of neurites within the voxel, and partial volume effects.^6,7^ Nevertheless, they provide added information and aid characterization of brain microstructure^8^ especially when considered along with NODDI metrics. For example, RD measures diffusivity perpendicular to the axons and thus increases with demyelination, whereas AD which measures diffusivity parallel to the axon and increases with edema but can decrease with debris due to axonal injury. MD provides a directionally averaged measure of diffusivity and is thus affected by both membrane density and fluid viscosity and is more interpretable in both white and gray matter. FA is a normalized measure of changes across the three principal diffusion axes and has been commonly used as a summary measure that is very sensitive to changes in microstructure^8^ but is affected by many factors. For example, for a given neurite density, higher ODI results in lower FA.^5,9^ Increases in FISO and the diffusivities (MD, RD, and AD) are associated with aging.^3^ Decreases in white matter FA values are also associated with aging,^3^ and have been found in asthma,^10^ but as noted previously, need to be interpreted in conjunction with changes in other dMRI metrics. Consideration of predicted changes in anatomy in the clinical sample, as well as examination of relationships with functional outcomes remain necessary for interpretation. For example, lower NDI in temporal and parietal cortical regions has been shown to differentiate mild cognitive impairment from controls, whereas lower ODI in frontal, temporal and parietal cortices was the differentiating factor in Alzheimer’s disease.^11^ Inverse relationships between NDI and amyloid and phosphorylated-tau burden have been consistently found, even in gray matter regions where no evidence of cortical thinning was found.^11,12^ Overall, the use of NODDI and DTI metrics in conjunction with dementia related CSF biomarkers in the present study provides a rich characterization of the impact of asthma on brain health.

## Discussion of additional findings

Apart from neurogranin, another biomarker associated with synaptic loss is α-synuclein.^13^ In gray matter, our finding of stronger positive relationships between FA and neurogranin as well as α-synuclein for individuals with asthma had a strong spatial overlap in the cerebellum, which is likely driven by the concomitant association with reductions in ODI, suggestive of reduced dendritic arborization.^5^ Although not directly related to synaptic degeneration, it is noteworthy that we previously observed an inverse relationship between plasma pTau_181_ and white matter MD in the cerebellum^14^; a region that merits closer attention in future studies examining the impact of asthma on brain health.

Within gray matter, we found that asthma accelerated the age-related increase in MD, RD, and AD, and to a lesser extent FISO. These findings are in line with reports that, although MD in gray matter is prone to partial volume effects, it is a sensitive marker of early neurodegenerative changes prior to significant brain atrophy.^15^

Our findings related to the influence of asthma on the relationship between brain microstructure and biomarkers of glial activation and neuroinflammation were complex and in line with reports of the changing nature of diverse neuroimmune interactions contributing to neurodegeneration^16^ and progression to dementia.^17^ In contrast to the relationships between brain microstructure and CSF concentrations of S100B and IL-6 that suggested a better profile in those with asthma, relationships between brain microstructure and YKL-40 as well as sTREM2 revealed evidence of an enhanced adverse effect of gliosis. YKL-40 is abundantly expressed in the brain by both microglia and astrocytes and its elevated presence in CSF has diagnostic and prognostic value in Alzheimer’s disease.^18^ Among aging CU individuals at risk of Alzheimer’s disease, baseline levels of YKL-40 have been associated with increased MD over time in the cingulum^19^ suggesting that the influence of asthma found in our study might be linked to altered glial activity or morphology. Further, we also found a stronger positive relationship between CSF sTREM2, a biomarker of microglial activation,^20^ and cerebellar gray matter FA in asthma that was spatially co-located with the stronger inverse relationship seen between neurogranin and ODI in asthma, suggesting that microglial activity might be contributing to synaptic pruning and reduced dendritic complexity. An important caveat is that the influence of asthma on the relationships with YKL-40 and sTREM2 was localized and in the absence of a significant omnibus test whereas the neuroprotective relationship with IL-6 was widespread and robust. We had previously reported strong and widespread positive relationships of plasma concentrations of the astrocytic marker GFAP with MD as well as with asthma severity in a large sample comprised of younger, CU individuals.^14^ The lack of a similar finding with CSF GFAP in the present study, plausibly due to lower power, was therefore unexpected and warrants further investigation. There is dynamicity in glial activation, proliferation and morphological alterations during acute inflammation, and glial cells are sensitive to, and interact with, other cell types in their neighbouring environments, which could contribute to the complex nature of glial responses seen in our study.^17,21^

## Supplementary Figures and Tables

All brain images are shown in radiological convention (left hemisphere is shown on the right side on coronal and axial views).


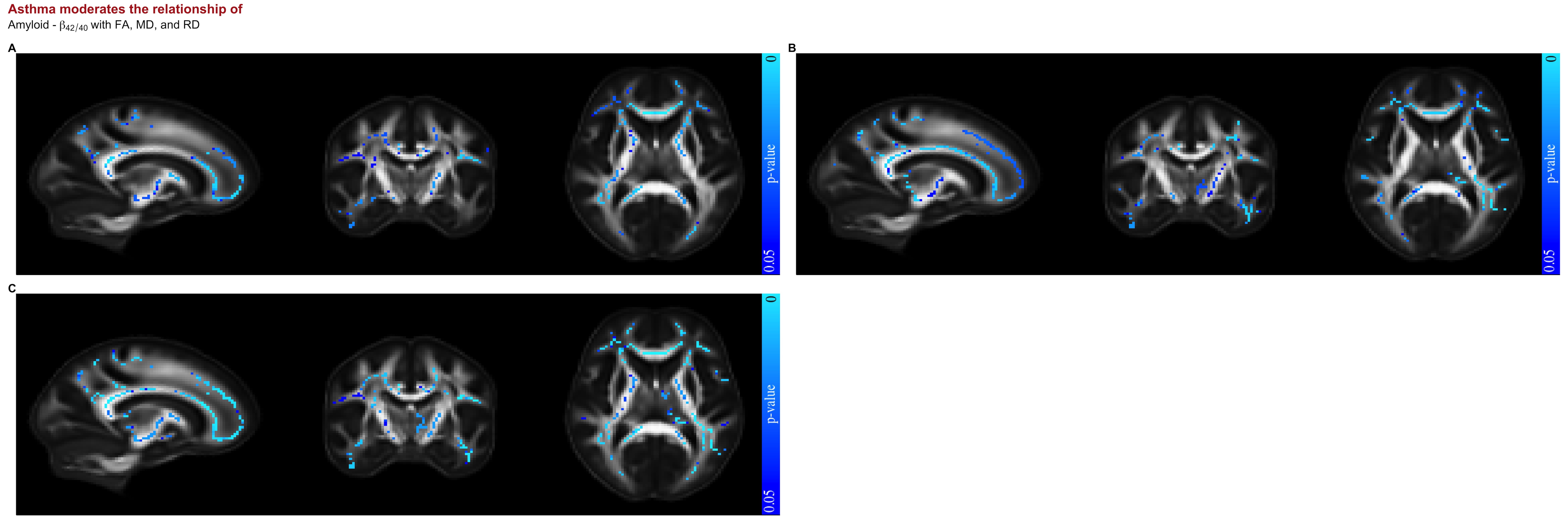


**Supplementary Figure 1. Asthma moderates the relationship between Aβ42/Aβ40** **and dMRI metrics.** Representative slices of white matter template displaying voxels where asthma significantly moderated (at *P* <.05, FWE corrected) the relationships between Aβ42/Aβ40 and FA (A), MD (B) as well as RD (C) in white matter.


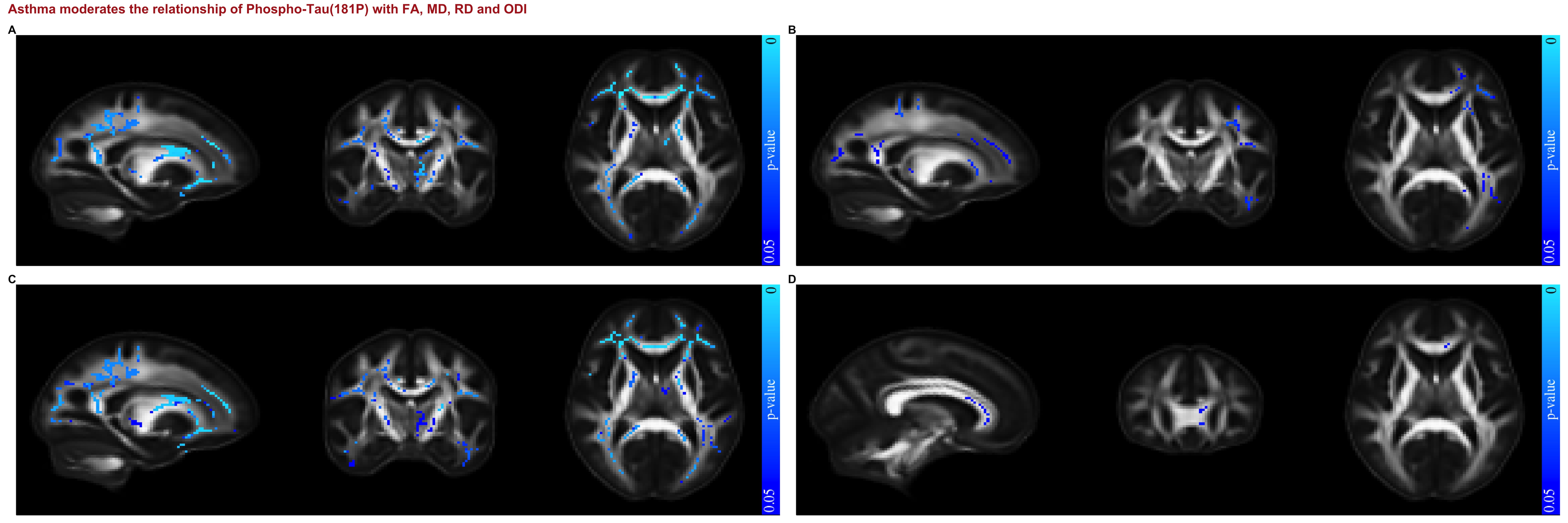


**Supplementary Figure 2. Asthma moderates the relationship between Phospho-tau(181P)** **and dMRI metrics.** Representative slices of white matter template displaying voxels where asthma significantly moderated (at *P* <.05, FWE corrected) the relationships between Phospho-tau(181P) and FA (A), MD (B), RD (C), and ODI (D) in white matter.


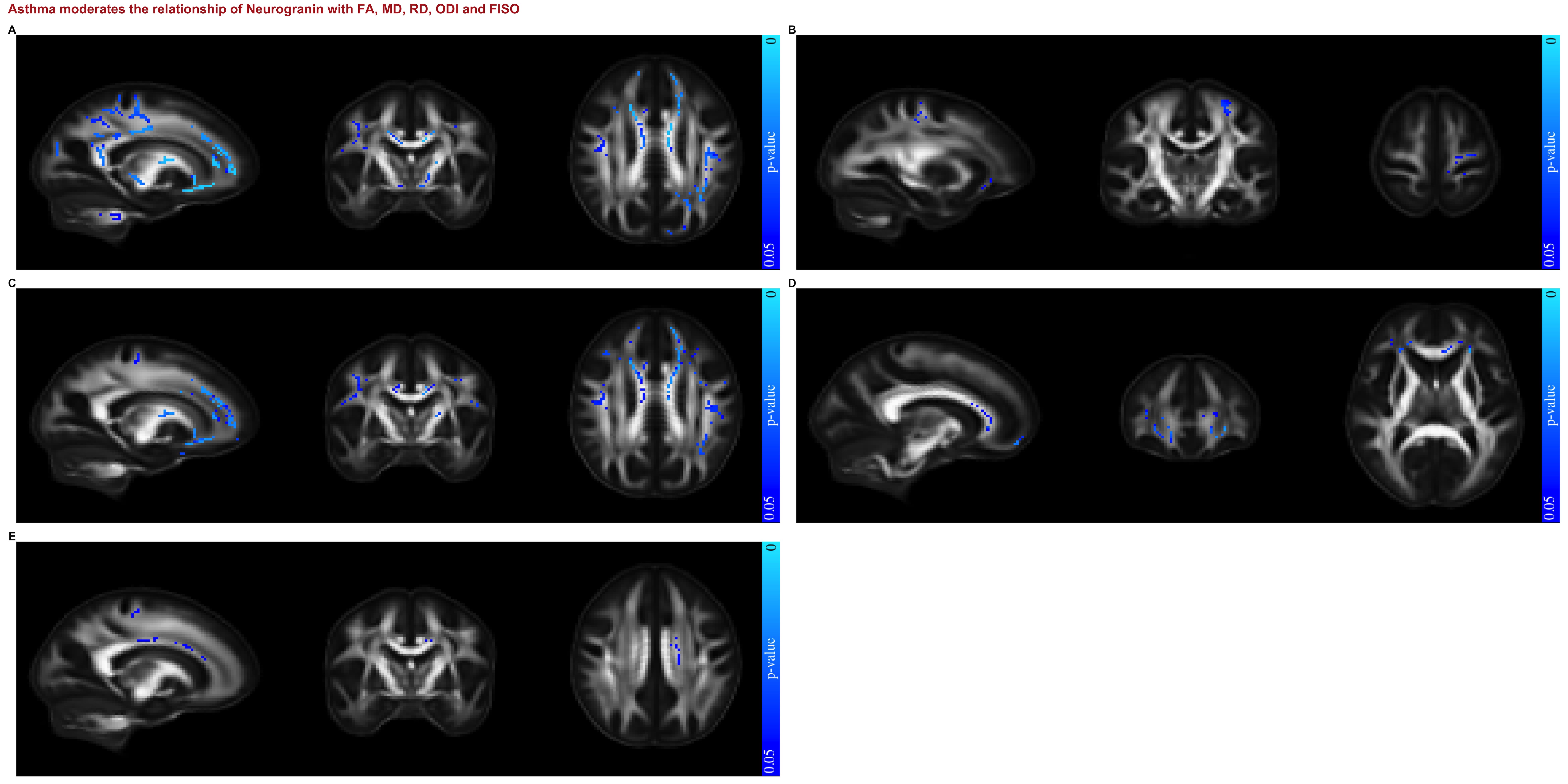


**Supplementary Figure 3. Asthma moderates the relationship between neurogranin** **and dMRI metrics.** Representative slices of white matter template displaying voxels where asthma significantly moderated (at *P* <.05, FWE corrected) the relationships between neurogranin and FA (A), MD (B), RD (C), ODI (D) and FISO (E) in white matter.


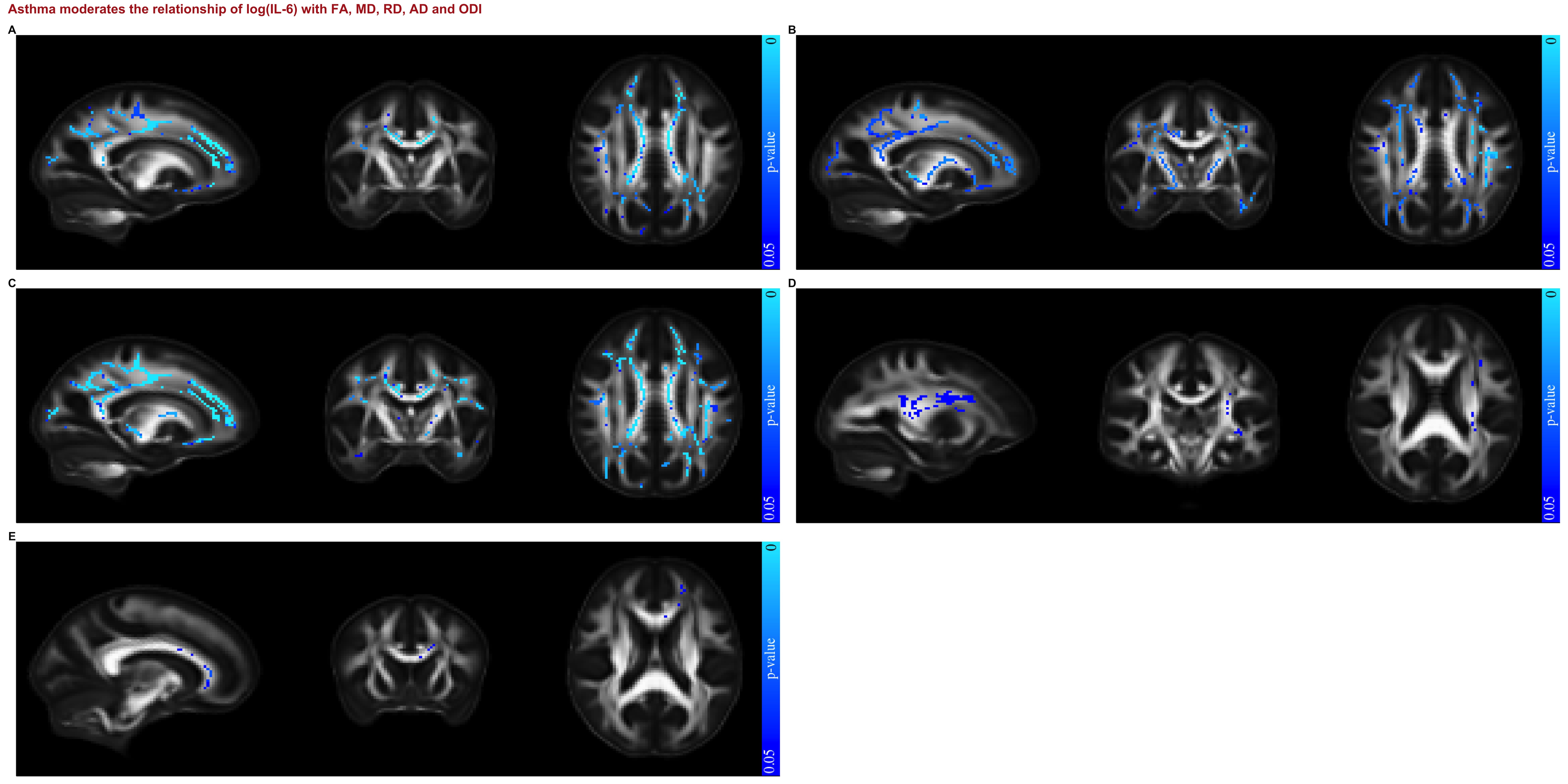


**Supplementary Figure 4. Asthma moderates the relationship between log (IL-6)** **and dMRI metrics.** Representative slices of white matter template displaying voxels where asthma significantly moderated (at *P* <.05, FWE corrected) the relationships between log (IL-6) and FA (A), MD (B), RD (C), AD (D) and ODI (E) in white matter.


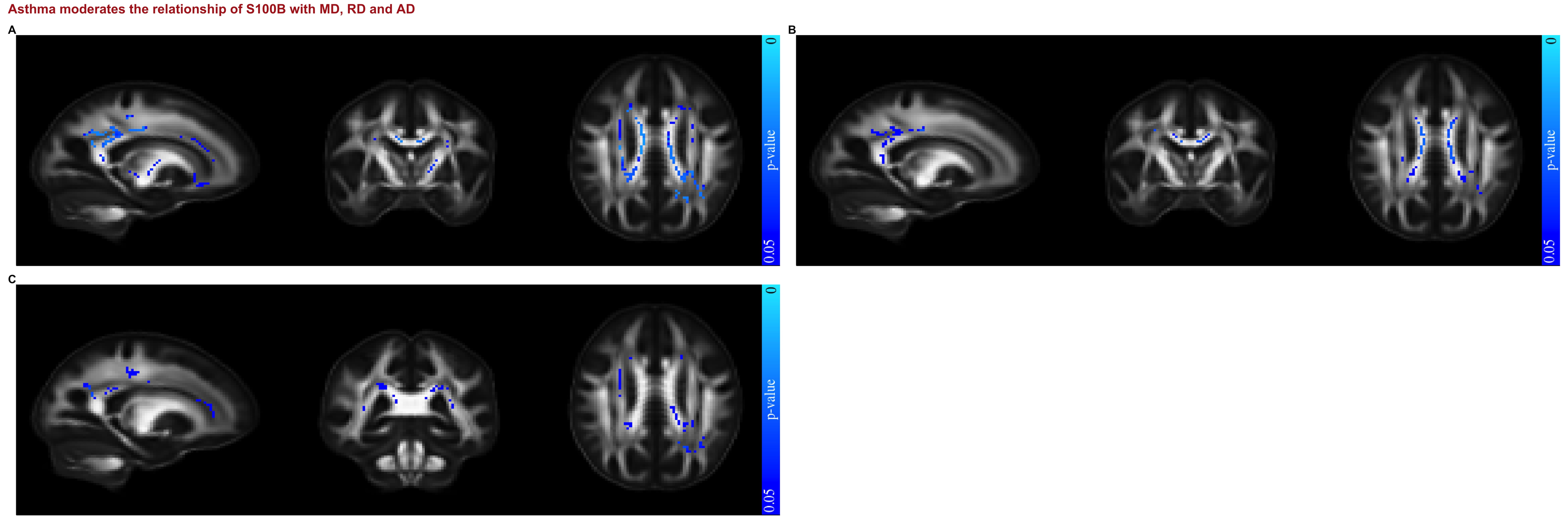


**Supplementary Figure 5. Asthma moderates the relationship between S100B** **and dMRI metrics.** Representative slices of white matter template displaying voxels where asthma significantly moderated (at *P* <.05, FWE corrected) the relationships between S100B and MD (A), RD (B) and AD (C) in white matter.


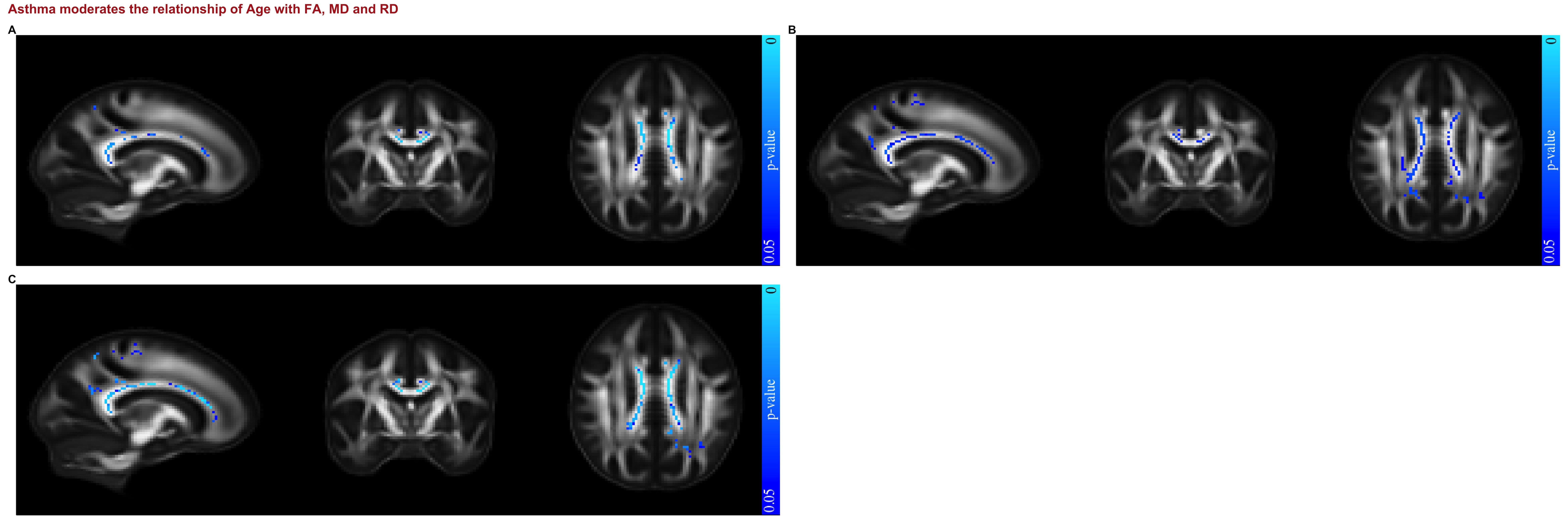


**Supplementary Figure 6. Asthma moderates the relationship between age** **and dMRI metrics.** Representative slices of white matter template displaying voxels where asthma significantly moderated (at *P* <.05, FWE corrected) the relationships between age and FA (A), MD (B), and RD (C) in white matter.

**
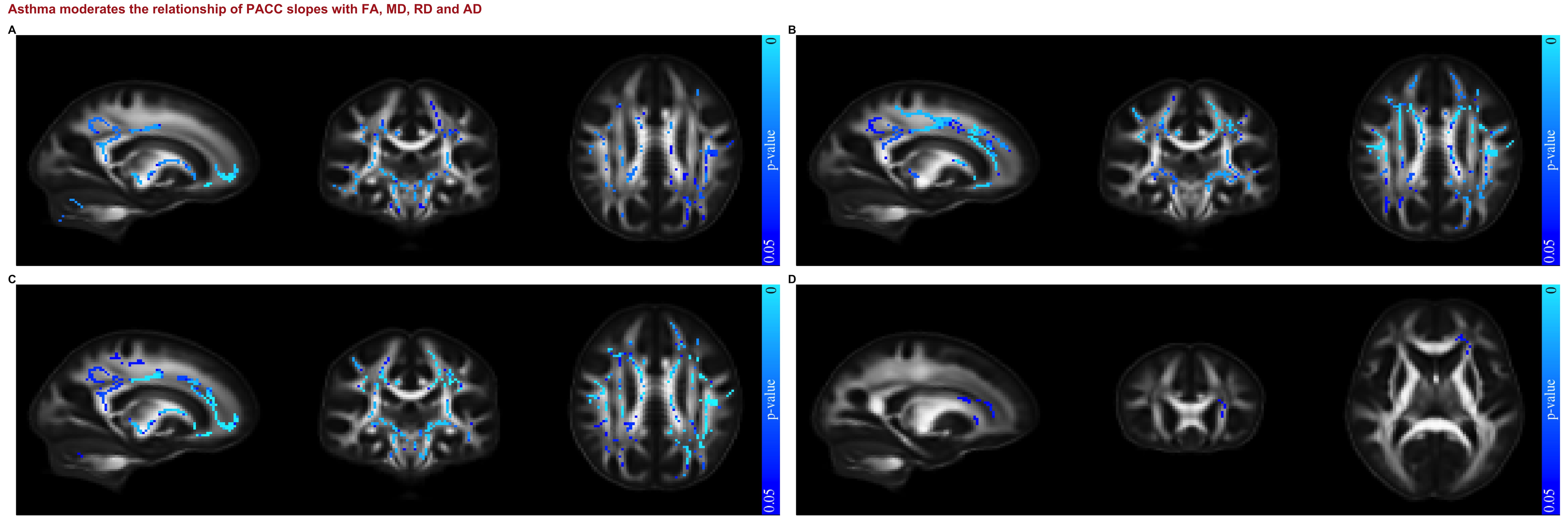
**

**Supplementary Figure 7. Asthma moderates the relationship between PACC slopes** **and dMRI metrics.** Representative slices of white matter template displaying voxels where asthma significantly moderated (at *P* <.05, FWE corrected) the relationships between PACC slopes and FA (A), MD (B), RD (C) and AD (D) in white matter.


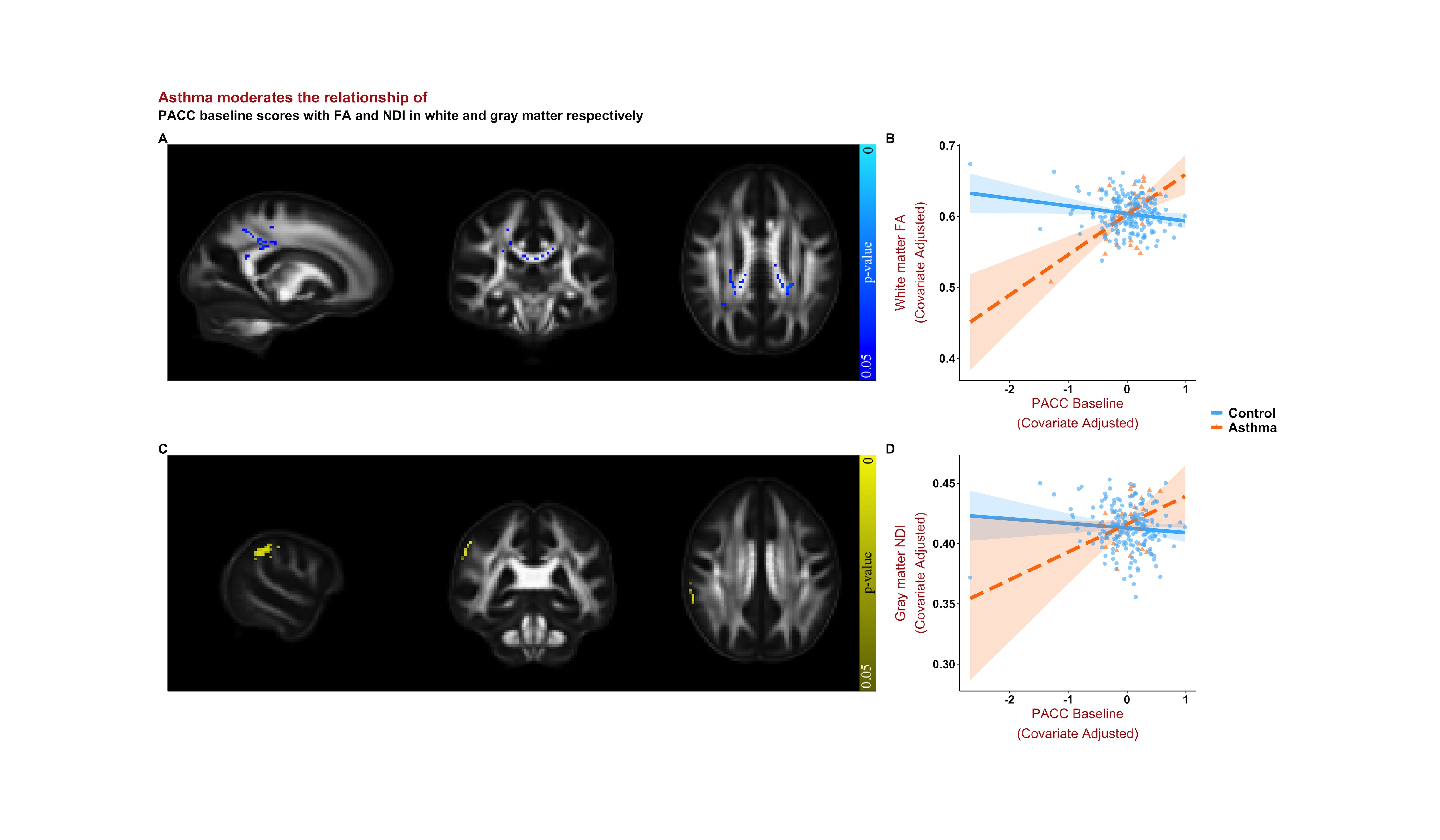


**Supplementary Figure 8. Asthma moderates the relationship of PACC scores at baseline (in WM and GM) with DWI metrics.** Representative slices of white matter and gray matter templates displaying voxels where asthma significantly moderated (at *P* <.05, FWE corrected) the relationships between PACC slopes and FA in white matter (median β = 0.338) (A), and NDI in gray matter (median β = 0.123) (C). Corresponding scatterplots (B and D) generated for visualization by extracting the mean of all significant voxels, with one data point for each individual, after controlling for age, sex and cognitive status. Regressions were carried out using PALM with omnibus correction across all seven dMRI metrics.

**Supplementary Table 1. Detailed results of the moderating effect of asthma.**

| **Additional covariates** | | |  |  |  |  |  |  |
| --- | --- | --- | --- | --- | --- | --- | --- | --- |
| **Category** | **Predictor** | **Brain Region** | **Direction** | **Main model** | **ASCVD** | ***APOE*4** | **ASCVD & *APOE*4** | |
| Group | Overall | WM | ↑ | ODI^ | ODI^ | ODI^ | ODI^ | |
| Alzheimer’s disease pathology | Aβ42/Aβ40^a^ | WM | ↑ | MD, RD | MD, RD | MD, RD | MD, RD | |
|  |  |  | ↓ | FA, NDI | FA, NDI | FA, NDI | FA, NDI | |
|  | Phospho-tau(181P) |  | ↑ | MD, RD, ODI | MD, RD, ODI | MD, RD, ODI | MD, RD, ODI | |
|  |  |  | ↓ | FA, NDI | FA, NDI | FA, NDI | FA, NDI | |
| Synaptic integrity | Neurogranin | WM | ↑ | FISO, MD, ODI, RD | FISO, ODI | FISO, ODI | FISO, ODI | |
|  |  |  | ↓ | FA, NDI |  | FA |  | |
|  |  | GM | ↑ | FA^ | FA^ | FA^ | FA^ | |
|  |  |  | ↓ | ODI^ | ODI^ | ODI^ | ODI^ | |
|  | α-synuclein | WM | ↑ | ODI^ |  |  |  | |
|  |  | GM | ↑ | FA^ | FA^ | FA^ | FA^ | |
| Neuroinflammation/ Glial activation | Log IL-6 | WM | ↑ | FA, NDI | FA, NDI | FA, NDI | FA, NDI | |
|  |  |  | ↓ | AD, MD, ODI, RD | MD, RD | MD, RD | MD, RD | |
|  | S100B |  | ↓ | AD, FISO, MD, RD |  |  |  | |
|  | YKL-40 |  | ↑ |  |  | FISO |  | |
|  |  | GM | ↑ |  | FISO^ | FISO^ | FISO^ | |
|  | sTREM2 |  | ↑ | FA^ | FA^ | FA^ |  | |
| Aging | Age | WM | ↑ | FISO, MD, RD | FISO^ | FISO^ | FISO^ | |
|  |  |  | ↓ | FA |  |  |  | |
|  |  | GM | ↑ | AD, MD, RD* |  | FISO^ | FISO^ | |
| Cognition | PACC slopes^a^ | WM | ↑ | AD, MD, RD | RD* | RD* | RD* | |
|  |  |  | ↓ | FA, NDI | FA, NDI* | FA, NDI* | FA, NDI* | |
|  |  | GM | ↑ | AD, MD, RD |  |  |  | |
|  |  |  | ↓ | NDI |  |  |  | |
|  | PACC baseline^a^ | WM | ↓ | FA |  |  |  | |
|  |  | GM | ↓ | NDI | NDI | NDI | NDI | |

**Notes:** PALM analyses revealed the moderating influence of asthma on associations between the predictors and a range of dMRI metrics in both WM and GM, controlling for age, sex, and cognitive status (cognitively unimpaired or not). The models for age controlled for sex and cognitive status. PACC scores were z-scored with respect to data from all participants at the ADRC. PACC slopes were derived from change of PACC scores across assessment visits; analyses were re-run separately and jointly controlling for ASCVD and *APOE*4 to ascertain if the influence of asthma was independent or overlapping with the effects of these known risk factors of neurodegeneration. All findings were significant at FWE-corrected *P* < 0.05. The omnibus test across all dMRI metrics, unless otherwise indicated, was also significant at FWE-corrected *P* < 0.05. Arrows in the “Direction” column indicate direction of the moderating influence of asthma for the dMRI metrics listed for each model.

^a^Direction of change for dMRI metrices for Aβ42/Aβ40, and PACC variables are reversed for easier understanding, as lower values indicate more adverse impact.

*Omnibus at FWE corrected trend *P* < 0.1.

^Omnibus not significant after FWE correction, *P* > 0.1.

**Abbreviations:** AD = axial diffusivity; ADRC = Wisconsin Alzheimer’s Disease Research Center; Aβ = β-amyloid; *APOE*4 = apolipoprotein E ε4 carrier; ASCVD = atherosclerotic cardiovascular disease 10-year risk; dMRI = diffusion MRI; FA = fractional anisotropy; FISO = isotropic volume fraction; FWE = familywise error; GM = gray matter; IL-6 = interleukin-6; MD = mean diffusivity; NDI = neurite density index; ODI = orientation dispersion index; PACC = preclinical Alzheimer cognitive composite; PALM = permutation analysis of linear models; Phospho-tau(181P) = phosphorylated-tau-181; RD = radial diffusivity; S100B = S100 calcium binding protein B; sTREM2 = soluble triggering receptor expressed on myeloid cells 2; WM = white matter; YKL-40 = chitinase-3-like protein 1.

# Supplementary References

1. Nazeri A, Schifani C, Anderson JAE, Ameis SH, Voineskos AN. In Vivo Imaging of Gray Matter Microstructure in Major Psychiatric Disorders: Opportunities for Clinical Translation. *Biol Psychiatry Cogn Neurosci Neuroimaging*. 2020;5(9):855-864. doi:10.1016/j.bpsc.2020.03.003

2. Motovylyak A, Vogt NM, Adluru N, et al. Age-related differences in white matter microstructure measured by advanced diffusion MRI in healthy older adults at risk for Alzheimer’s disease. *Aging Brain*. 2022;2:100030. doi:10.1016/j.nbas.2022.100030

3. Billiet T, Vandenbulcke M, Mädler B, et al. Age-related microstructural differences quantified using myelin water imaging and advanced diffusion MRI. *Neurobiol Aging*. 2015;36(6):2107-2121. doi:10.1016/j.neurobiolaging.2015.02.029

4. Yi SY, Barnett BR, Torres-Velázquez M, et al. Detecting microglial density with quantitative multi-compartment diffusion MRI. *Front Neurosci*. 2019;13(FEB):1-9. doi:10.3389/fnins.2019.00081

5. Zhang H, Schneider T, Wheeler-Kingshott CA, Alexander DC. NODDI: Practical in vivo neurite orientation dispersion and density imaging of the human brain. *Neuroimage*. 2012;61(4):1000-1016. doi:10.1016/j.neuroimage.2012.03.072

6. Jones DK, Knösche TR, Turner R. White matter integrity, fiber count, and other fallacies: The do’s and don’ts of diffusion MRI. *Neuroimage*. 2013;73:239-254. doi:10.1016/j.neuroimage.2012.06.081

7. Jones DK, Cercignani M. Twenty-five pitfalls in the analysis of diffusion MRI data. *NMR Biomed*. 2010;23(7):803-820. doi:10.1002/nbm.1543

8. Alexander AL, Hurley SA, Samsonov AA, et al. Characterization of Cerebral White Matter Properties Using Quantitative Magnetic Resonance Imaging Stains. *Brain Connect*. 2011;1(6):423-446. doi:10.1089/brain.2011.0071

9. Grussu F, Schneider T, Tur C, et al. Neurite dispersion: a new marker of multiple sclerosis spinal cord pathology? *Ann Clin Transl Neurol*. 2017;4(9):663-679. doi:10.1002/acn3.445

10. Bian R, Zhang Y, Yang Y, et al. White Matter Integrity Disruptions Correlate With Cognitive Impairments in Asthma. *J Magn Reson Imaging*. 2018;48(3):748-756. doi:10.1002/jmri.25946

11. Vogt NM, Hunt JF, Adluru N, et al. Cortical Microstructural Alterations in Mild Cognitive Impairment and Alzheimer’s Disease Dementia. *Cereb Cortex*. 2020;30(5):2948-2960. doi:10.1093/cercor/bhz286

12. Parker TD, Slattery CF, Zhang J, et al. Cortical microstructure in young onset Alzheimer’s disease using neurite orientation dispersion and density imaging. *Hum Brain Mapp*. 2018;39(7):3005-3017. doi:10.1002/hbm.24056

13. Blennow K, Zetterberg H. The Past and the Future of Alzheimer’s Disease Fluid Biomarkers. *J Alzheimer’s Dis*. 2018;62(3):1125-1140. doi:10.3233/JAD-170773

14. Rosenkranz MA, Dean DC, Bendlin BB, et al. Neuroimaging and biomarker evidence of neurodegeneration in asthma. *J Allergy Clin Immunol*. 2022;149(2):589-598.e6. doi:10.1016/j.jaci.2021.09.010

15. Henf J, Grothe MJ, Brueggen K, Teipel S, Dyrba M. Mean diffusivity in cortical gray matter in Alzheimer’s disease: The importance of partial volume correction. *NeuroImage Clin*. 2018;17(September 2017):579-586. doi:10.1016/j.nicl.2017.10.005

16. Sankowski R, Mader S, Valdés-Ferrer SI. Systemic inflammation and the brain: Novel roles of genetic, molecular, and environmental cues as drivers of neurodegeneration. *Front Cell Neurosci*. 2015;9(FEB):1-20. doi:10.3389/fncel.2015.00028

17. Haage V, De Jager PL. Neuroimmune contributions to Alzheimer’s disease: a focus on human data. *Mol Psychiatry*. 2022;(May):1-18. doi:10.1038/s41380-022-01637-0

18. Dhiman K, Blennow K, Zetterberg H, Martins RN, Gupta VB. Cerebrospinal fluid biomarkers for understanding multiple aspects of Alzheimer’s disease pathogenesis. *Cell Mol Life Sci*. 2019;76(10):1833-1863. doi:10.1007/s00018-019-03040-5

19. Racine AM, Merluzzi AP, Adluru N, et al. Association of longitudinal white matter degeneration and cerebrospinal fluid biomarkers of neurodegeneration, inflammation and Alzheimer’s disease in late-middle-aged adults. *Brain Imaging Behav*. 2019;13(1):41-52. doi:10.1007/s11682-017-9732-9

20. Suárez‐Calvet M, Kleinberger G, Araque Caballero MÁ, et al. sTREM 2 cerebrospinal fluid levels are a potential biomarker for microglia activity in early‐stage Alzheimer’s disease and associate with neuronal injury markers . *EMBO Mol Med*. 2016;8(5):466-476. doi:10.15252/emmm.201506123

21. Garcia-Hernandez R, Cerdán Cerdá A, Trouve Carpena A, et al. Mapping microglia and astrocyte activation in vivo using diffusion MRI. *Sci Adv*. 2022;8(21):2020.02.07.938910. doi:10.1126/sciadv.abq2923
